# Identification of Bacterial Metabolites Modulating Breast Cancer Cell Proliferation and Epithelial-Mesenchymal Transition

**DOI:** 10.3390/molecules28155898

**Published:** 2023-08-05

**Authors:** Gyula Ujlaki, Tünde Kovács, András Vida, Endre Kókai, Boglára Rauch, Szandra Schwarcz, Edit Mikó, Eszter Janka, Adrienn Sipos, Csaba Hegedűs, Karen Uray, Péter Nagy, Peter Bai

**Affiliations:** 1 Department of Medical Chemistry, Faculty of Medicine, University of Debrecen, 4032 Debrecen, Hungary; ujlaki.gyula@med.unideb.hu (G.U.); kovacs60.tunde90@gmail.com (T.K.); avida@foss.dk (A.V.); ekokai@med.unideb.hu (E.K.); rauch.boglarka@med.unideb.hu (B.R.); schwarcz.szandra@med.unideb.hu (S.S.); miko.edit@med.unideb.hu (E.M.); siposadri@med.unideb.hu (A.S.); hcsaba@med.unideb.hu (C.H.); karen.uray@med.unideb.hu (K.U.); 2 Department of Dermatology, Faculty of Medicine, University of Debrecen, 4032 Debrecen, Hungary; janka.eszter.a@gmail.com; 3 Department of Biophysics and Cell Biology, Faculty of Medicine, University of Debrecen, 4032 Debrecen, Hungary; nagyp@med.unideb.hu; 4 MTA-DE Lendület Laboratory of Cellular Metabolism, 4032 Debrecen, Hungary; 5 Research Center for Molecular Medicine, Faculty of Medicine, University of Debrecen, 4032 Debrecen, Hungary; 6 ELKH-DE Cell Biology and Signaling Research Group ELKH, 4032 Debrecen, Hungary

**Keywords:** high content screening, butyric acid, vanillic acid, glycolic acid, d-mannitol, trans-ferulic acid, 2,3-butanediol, 4-hydroxybenzoic acid, hydrocinnamic acid, 3-hydroxyphenylacetic acid, epithelial-mesenchymal transition, proliferation, bacterial metabolite, dysbiosis, breast cancer, metabolite signaling, microbiome, secretome

## Abstract

Breast cancer patients are characterized by the oncobiotic transformation of multiple microbiome communities, including the gut microbiome. Oncobiotic transformation of the gut microbiome impairs the production of antineoplastic bacterial metabolites. The goal of this study was to identify bacterial metabolites with antineoplastic properties. We constructed a 30-member bacterial metabolite library and screened the library compounds for effects on cell proliferation and epithelial-mesenchymal transition. The metabolites were applied to 4T1 murine breast cancer cells in concentrations corresponding to the reference serum concentrations. However, yric acid, glycolic acid, d-mannitol, 2,3-butanediol, and trans-ferulic acid exerted cytostatic effects, and 3-hydroxyphenylacetic acid, 4-hydroxybenzoic acid, and vanillic acid exerted hyperproliferative effects. Furthermore, 3-hydroxyphenylacetic acid, 4-hydroxybenzoic acid, 2,3-butanediol, and hydrocinnamic acid inhibited epithelial-to-mesenchymal (EMT) transition. We identified redox sets among the metabolites (d-mannitol—d-mannose, 1-butanol—butyric acid, ethylene glycol—glycolic acid—oxalic acid), wherein only one partner within the set (d-mannitol, butyric acid, glycolic acid) possessed bioactivity in our system, suggesting that changes to the local redox potential may affect the bacterial secretome. Of the nine bioactive metabolites, 2,3-butanediol was the only compound with both cytostatic and anti-EMT properties.

## 1. Introduction

Breast cancer is the most frequent cancer in women [1,2]. Breast cancer is characterized by the disturbance of multiple microbiome compartments, termed oncobiosis [3]. Oncobiosis affects the microbiomes of breast tissue [4,5], milk ducts [6], the tumor’s inherent microbiome [7,8,9,10,11,12,13,14,15,16,17,18,19,20,21], the distal gut [22,23,24,25,26,27,28,29,30,31,32,33,34,35,36,37,38,39,40,41,42], and the urinary tract [13,43]. Oncobiosis support of breast tumors is multifaceted. (1) Bacteria colonize the tumor tissue; (2) the immune system favors the tolerogenic state [44]; and (3) the gut microbiome’s capacity to produce antitumor metabolites is suppressed [29,30,31,45,46,47].

The gut microbiome has a large capacity to produce a diverse set of bioactive metabolites. These low-molecule-weight biomolecules can enter the systemic circulation and reach different cell types in the human body, including cancer cells. This process is similar to the action of hormones or paracrine signaling molecules. The first such “metabolite” was identified more than 50 years ago [48] using the observation that the gut oncobiome in breast cancer has a larger capacity to reactivate conjugated estrogens via beta-glucuronidases than the gut eubiome. Multiple cytostatic metabolites produced by the gut eubiome have been identified in breast cancer, including lithocholic acid [29,49], cadaverine [30], indole metabolites [31,32], and short-chain fatty acids [50,51]. These metabolites exert antineoplastic effects, including modulation of cell proliferation and epithelial-mesenchymal transition (EMT) [44]. As stated earlier, the metabolic capacity of the microbiome is suppressed in breast cancer, and the capacity to produce known antineoplastic metabolites decreases in breast cancer [29,30,31,45,46,47].

Given the enormous and diverse metabolic capacity of the (gut) microbiome, other bacterial metabolites may also possess biological activity toward breast cancer cells. The goal of this study was to identify bacterial metabolites with antineoplastic activities in breast cancer.

## 2. Results

### 2.1. Metabolite Library Construction

Bacterial metabolites that interfere with human metabolism were identified via a literature search. The metabolites identified using this search were curated to eliminate falsely identified metabolites (i.e., not produced in bacteria). The serum reference concentration of the metabolites was established using literature searches (see Table 1).

A recent report [82] showed that tumors impact gut motility by modulating beta-adrenergic receptors. Varying the speed of passage likely changes the redox environment of the GI tract [83], suggesting that the redox state of redox-labile metabolites may change in breast cancer patients. Therefore, the redox partner of the applicable metabolites (mannose—d-mannitol, TMA—TMAO, 1-butanol—butyric acid, ethylene glycol—glycolic acid, oxalic acid) was introduced into the library, and the serum reference concentrations were established using a literature search. The literature search did not yield results for ethylene glycol; therefore, the same concentration was used as the redox partner with a known reference concentration. The metabolites, serum reference concentrations, treatment concentrations, and corresponding literature are listed in Table 1.

### 2.2. Development of High-Throughput Methods for Assessing Cell Proliferation

In previous studies, we identified and characterized a set of bacterial metabolites with cytostatic activity [29,30,31,32]; however, the antiproliferative activity of the metabolites was limited, and the SRB proliferation assay was not ideal for obtaining high-precision data under these conditions. Therefore, we tested two Image-analysis-based nuclei counting methods. Image segmentation and nuclei counting were performed using CellProfiler in one method, and segmentation was performed using a deep learning (DL)-based method developed by us, followed by nuclei counting using CellProfiler in the second method. 

**Figure 1:**
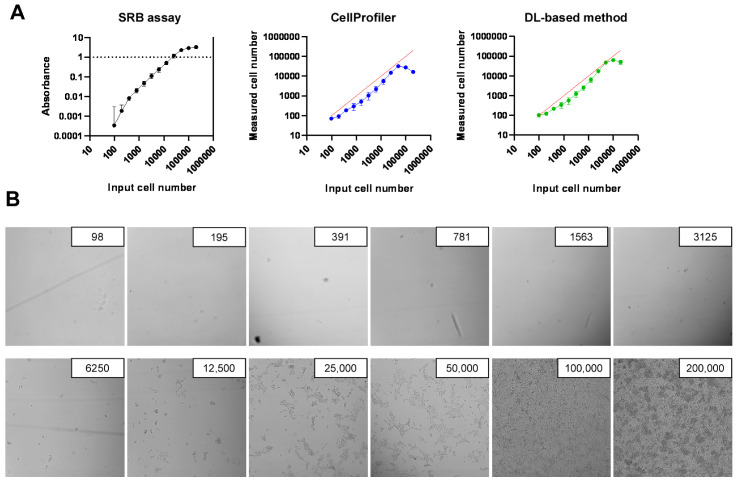
Validation of the methods used to assess cell proliferation. (**A**) The indicated number of cells were plated in 96-well plates for the SRB assay or PerkinElmer CellCarrier Ultra 96-well plates for the two high-content screening methods. On the following day, either the SRB assay or high-content screening method was conducted. For high-content screening, the nuclei were stained with DAPI, images were segmented using CellProfiler or the deep learning-based method, and nuclei numbers were counted using CellProfiler. The red lines indicate a 45° line, indicating the same number of cells as seeded. (**B**) Representative phase-contrast images of the wells containing the indicated number of cells. Abbreviations: DL—deep learning.

The SRB assay was unreliable at low cell densities in the range of 100–300 cells/well. Furthermore, due to the inherent limitations of photometry, values above an absorbance of 1.0 are not reliable, limiting the maximum number of cells that can be measured to 10,000–30,000 cells (Figure 1A). Values above an absorbance of 1.0 can be measured after dilution, which may introduce errors. Image-analysis-based assays have a larger dynamic range than the SRB assay. Both image-analysis-based assays were reliable for measuring low cell numbers (Figure 1A). The analysis-based assays were also able to detect up to 100,000 cells/well. On the high end of the scale, the DL-based method was superior to the CellProfiler’s built-in method (Figure 1A). In contrast to conventional methods, semantic segmentation models do not require parameter adjustment for every run. Of note, the image-analysis-based assays identified fewer cells than introduced if the number of plated cells was 1000–10,000. Visual inspection of the wells revealed that, at these densities, cells adhered less to plates, leading to lower cell numbers. Hence, cell numbers did indeed decrease in the culture plates (Figure 1B), suggesting that the image-analysis-based assays are precise. Based on these results, we applied the image-analysis-based assays in subsequent experiments.

### 2.3. Identification of Bacterial Metabolites with Pro and Antiproliferative Properties

**Figure 2:**
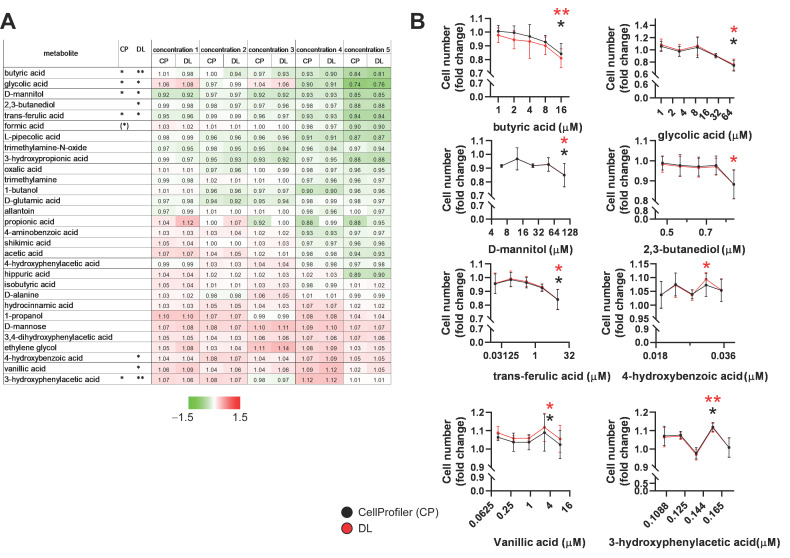
Identification of cytostatic bacterial metabolites in 4T1 breast cancer cells. The cells (800 4T1 cells/well) were plated in PerkinElmer CellCarrier Ultra 96-well plates and treated with the indicated metabolites at the specified concentrations for 48 h. Measurements were repeated at least three times using four technical replicates. Images were acquired and segmented using CellProfiler or the DL algorithm. Nuclei were counted in segmented images using CellProfiler. Data are represented as averages ± SDs of biological replicates. Values were normalized to vehicle-treated cells and expressed as fold changes. Each metabolite was statistically analyzed separately. (**A**) A heatmap representation of the effects of the metabolites on 4T1 cell proliferation. (**B**) The effects of the metabolites on 4T1 cell proliferation. Metabolite concentrations are displayed on the logarithmic axes. * and ** indicate statistically significant differences between vehicle-treated cells (control) and cells treated with the metabolite at *p* < 0.05 and *p* < 0.01, respectively. Abbreviations: DL—deep learning segmentation, CP—CellProfiler’s built-in method segmentation.

The capacity of the bacterial metabolites from the molecule library to modulate the proliferation of 4T1 breast cancer cells was assessed. Cells were treated with five metabolite concentrations spanning the reference concentrations listed in Table 1. Statistical analyses of the dose–response curves were performed independently. We identified seven bioactive metabolites: butyric acid, glycolic acid, d-mannitol, 2,3-butanediol, trans-ferulic acid, 4-hydroxybenzoic acid, vanillic acid, and 3-hydroxyphenylacetic acid (Figure 2). Formic acid displayed antiproliferative properties at the highest concentration; however, the highest concentration of formic acid considerably exceeded the upper end of the reference range (therefore, in Figure 1A, the significance symbol for formic acid is between brackets). However, yric acid, glycolic acid, d-mannitol, 2,3-butanediol, and trans-ferulic acid were antiproliferative at the highest applied concentrations, while 4-hydroxybenzoic acid, vanillic acid, and 3-hydroxyphenylacetic acid supported proliferation at specific concentrations (Figure 2B). 

### 2.4. Development of a High-Throughput Method for Assessing Epithelial-to-Mesenchymal Transition

**Figure 3:**
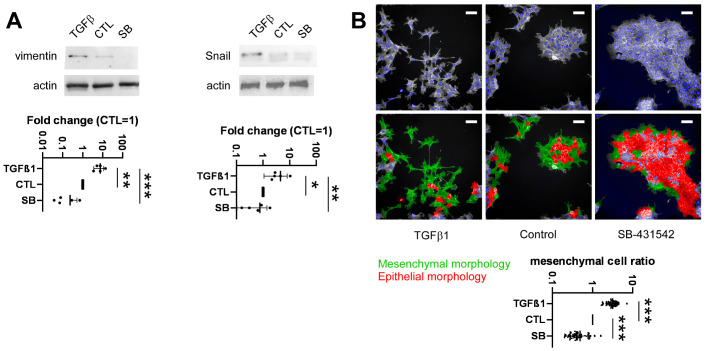
High-content screening-based methods can detect changes in cell morphology and indicate EMT. (**A**) 1.5 × 10^6^ 4T1 cells were plated in Petri dishes and treated with TGFβ or SB-431542 for 48 h. Cellular proteins were assessed using Western blotting with the indicated antibodies. Sample blots are shown along with their densitometric evaluation presented as means ± SD. Values were normalized to vehicle-treated (control) cells. (**B**) 800 4T1 cells/well were plated in PerkinElmer CellCarrier Ultra 96-well plates and treated with TGFβ or SB-431542 for 48 h. Nuclei were stained with DAPI, and cells were visualized using Texas Red-X Phalloidin. The images were segmented using the Harmony software (version 3.1.8) (PerkinElmer, Waltham, MA, USA) to identify cells with epithelial or mesenchymal morphology. Proportions of mesenchymal cells were normalized to total cell number and for inter-sample cell number differences. Normality was tested, and statistical significance was calculated as described in the Methods. Representative fluorescence microscopy images are presented. The scale bar equals 10 µm. *, **, and *** indicate statistically significant differences between vehicle-treated (control) cells and treated cells at *p* < 0.05, *p* < 0.01, and *p* < 0.001, respectively. Abbreviations: CTL—control, SB—SB-431542, TGFβ—transforming growth factor β.

The bacterial metabolites previously identified in breast cancer (lithocholic acid, cadaverine, indole derivatives, and short-chain fatty acids [44]) affected EMT. Therefore, we assessed the effects of members of the metabolite library on EMT. The high-content image analysis methodology was based on the observation that EMT changes cellular morphology in breast cancer [29,84,85,86]. In parallel, we assessed the protein levels of EMT markers for validation [29,84,85,86]. As a positive control for the induction of mesenchymal transition, transforming growth factor β (TGFβ 10 ng/mL final concentration for 48 h) was applied [87]. As a negative control (inhibition of EMT), we applied SB-431542 (2 µM final concentration for 48 h), an inhibitor of TGFβ superfamily type I activating receptor-like kinases [88]. The protein expression of mesenchymal transition markers (Zinc finger protein SNAI1 (SnaiI) and vimentin) increased in response to TGFβ, while vimentin, but not Snail, decreased in response to SB-431542 treatment in 4T1 cells (Figure 3A). Furthermore, visual inspection of the cells supported the TGFβ-induced mesenchymal and SB-431542-mediated epithelial transition (Figure 3B). In good agreement with these results, TGFβ induced increased proportions of mesenchymal cells, while SB-431542 decreased the proportions of mesenchymal cells (Figure 3B), as shown by both high-content screening-based methods. Based on these results, we concluded that the high-content screening-based methods reliably assessed the proportions of epithelial and mesenchymal cells under these conditions.

### 2.5. Identification of Bacterial Metabolites Modulating EMT

**Figure 4:**
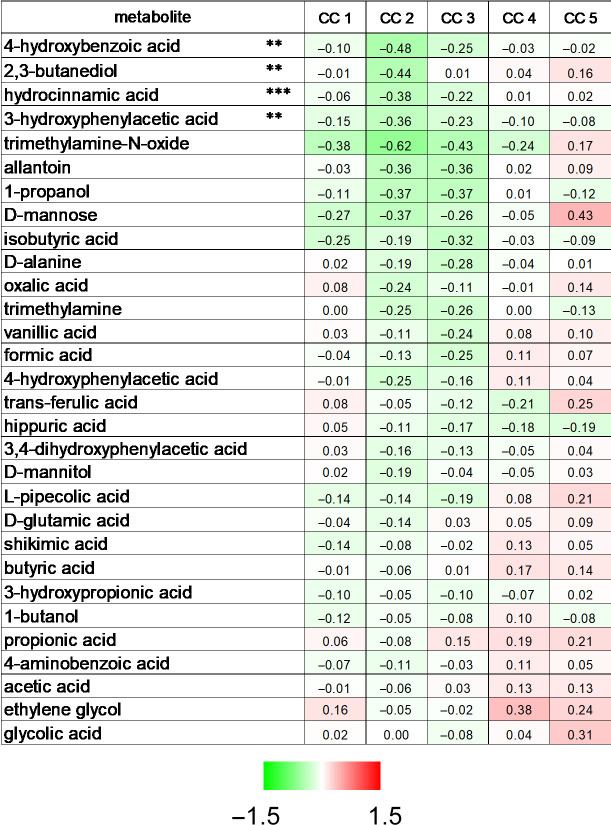
Identification of bioactive bacterial metabolites that suppress EMT. 800 4T1 cells/well were seeded in PerkinElmer CellCarrier Ultra 96-well plates and treated with the indicated metabolites at the concentrations specified in Table 1 for 48 h. The acquired images were segmented using the Harmony software (version 3.1.8) (PerkinElmer) to identify cells with epithelial or mesenchymal morphology. Proportions of the mesenchymal cells were normalized to total cell numbers within a sample and to inter-sample cell number differences. Normality was assessed, and statistical significance was calculated as described in the Methods. Each metabolite was statistically analyzed separately. ** and *** indicate statistically significant differences between vehicle-treated (control) cells and cells treated with a compound at *p* < 0.01 and *p* < 0.001, respectively. Abbreviations: CC 1–5—concentrations indicated in Table 1, the number references increasing doses.

**Figure 5:**
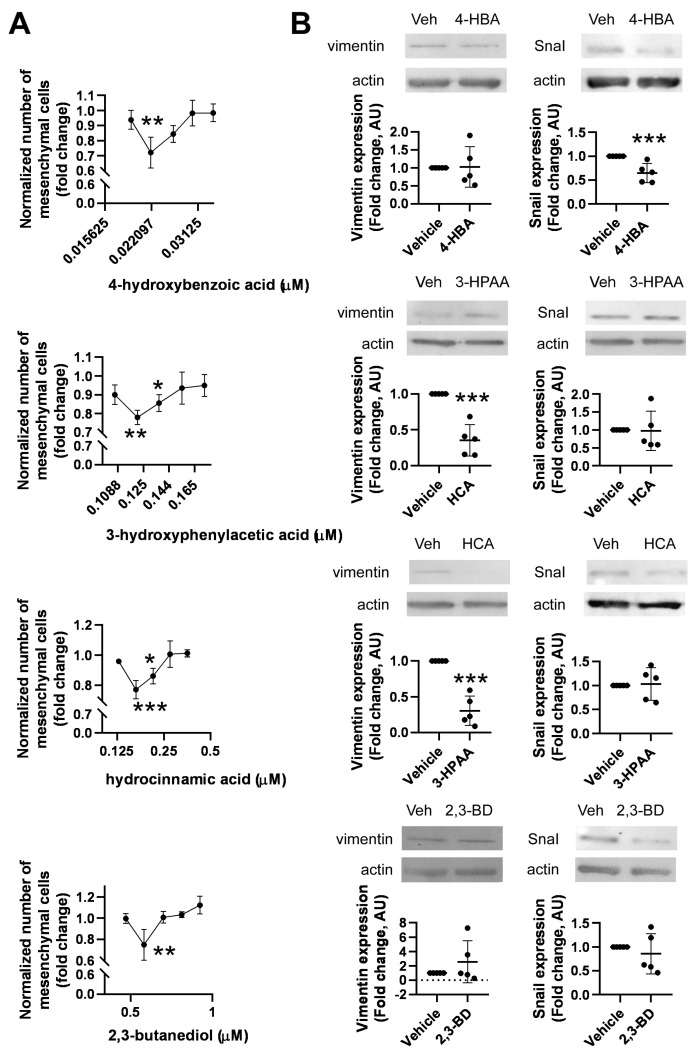
Identification of bioactive bacterial metabolites that suppress EMT. (**A**) 800 4T1 cells were plated on PerkinElmer CellCarrier Ultra 96-well plates and treated with the indicated metabolites at the concentrations specified in Table 1 for 48 h. The acquired images were segmented using the Harmony software (version 3.1.8) (PerkinElmer) to identify cells with epithelial or mesenchymal morphology. Proportions of the mesenchymal cells were normalized to total cell number within a sample and to inter-sample cell number differences. Normality was assessed, and statistical significance was calculated as described in the Methods. Each metabolite was statistically analyzed separately. (**B**) 1.5 × 10^6^ 4T1 cells were plated in Petri dishes and treated with the indicated metabolites at the concentrations specified in Table 1 for 48 h. Cellular proteins were assessed using Western blotting with the indicated antibodies. Sample blots and densitometry are shown (mean ± SD). Values were normalized to vehicle-treated (control) cells. *, **, and *** indicate statistically significant differences between vehicle-treated (control) cells and cells treated with a compound at *p* < 0.05, *p* < 0.01, and *p* < 0.001, respectively.

Members of the metabolite library were assessed for EMT effects using high-content screening. We identified 3-hydroxyphenylacetic acid, hydrocinnamic acid, 2,3-butanediol, and 4-hydroxybenzoic acid (Figure 4). In all cases, we observed a V-shape curve, where an optimal concentration of the compound significantly reduced the ratio of mesenchymal cells, but lower and higher concentrations were ineffective (Figure 5A). We verified these observations with Western blots. The 4T1 cells were treated with the effective concentration of the metabolites for 48 h, and the protein extracts were probed with anti-SnaiI and vimentin antibodies. All metabolites, except 2,3-butanediol, reduced the expression of one mesenchymal marker, validating the results of the high-content screening method (Figure 5B). For 2,3-butanediol, we observed a downward trend in the expression of the EMT markers (Figure 5B).

## 3. Discussion

Bacterial metabolite signaling plays an important role in regulating breast cancer progression [44]. Bioactive bacterial metabolites and toxins elicit multiple effects on breast cancer cells, including DNA instability, changes to cancer cell metabolism, EMT, cell movement, cancer stem cell proportions, cell proliferation, metastasis formation, and modulation of antitumor immune responses (reviewed in [44]). Most bacterial metabolites and toxins are synthesized by the gut microbiome; however, the tumor microbiome may also affect local metabolite and toxin levels (e.g., colibactin [4]). Most bacterial metabolites have antineoplastic features, and enzyme levels responsible for the biosynthesis of these metabolites are lower in breast cancer patients compared to controls (reviewed in [44]). 

**Figure 6:**
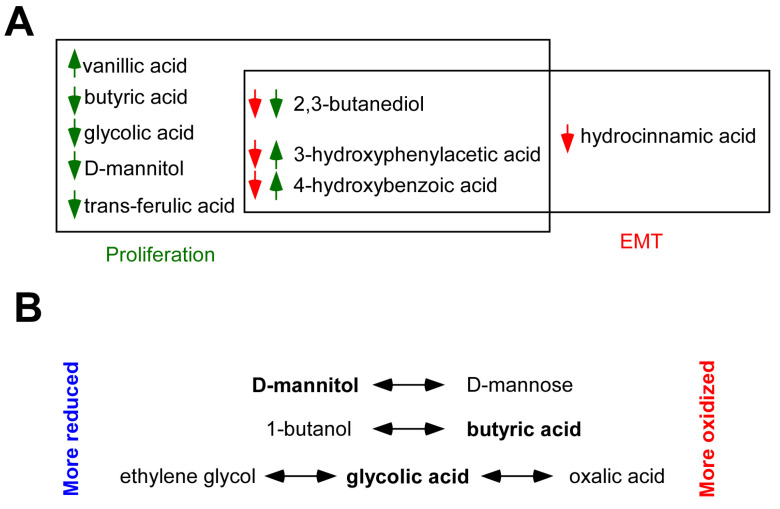
The biological and redox properties of bioactive bacterial metabolites were identified in this study. (**A**) Venn diagram of the bioactive metabolites highlighting their effects on cell proliferation and EMT. The arrows indicate the direction of changes (up—enhancement, down—inhibition); the green arrows indicate effects on cell proliferation, and the red arrows represent effects on EMT. (**B**) The schematic representation of the possible effects of the redox potential of the environment on the antiproliferative activity of the metabolites.

In this study, we identified nine bioactive metabolites that modulate cell proliferation and inhibit EMT, including butyric acid, vanillic acid, glycolic acid, d-mannitol, trans-ferulic acid, 2,3-butanediol, 4-hydroxybenzoic acid, hydrocinnamic acid, and 3-hydroxyphenylacetic acid (Figure 6A). The chemical nature of the metabolites is diverse, similar to their biochemistry and receptors. Of note, we applied these metabolites in concentrations corresponding to the physiological serum concentrations. The applied concentrations were in the low micromolar or submicromolar range; therefore, it is likely that the data presented here reflect the status of tumors.

Butyric acid and glycolic acid can be classified as short-chain fatty acids. However, yric acid plays multiple roles in breast cancer cells, including inhibitors of histone deacetylases (epigenetic modulators), metabolic substrates, and ligands of free fatty acid receptors (reviewed in [44]. However, yric acid levels decrease in the feces of breast cancer patients [41]. 

d-mannitol is a polyol, while 2,3-butanediol is a diol. The capacity for 2,3-butanediol synthesis is widespread among bacteria [89,90]. Importantly, 2,3-butanediol suppresses cell proliferation and inhibits EMT. Hence, this compound has the best antineoplastic profile among those assessed in this study. 

Interestingly, among short-chain fatty acids and polyols, multiple redox metabolite pairs were identified (**d-mannitol**—d-mannose, **1-butanol**—butyric acid, ethylene glycol—**glycolic acid**—oxalic acid; the active compounds are in bold); of which only one partner had cytostatic properties (Figure 6B). These data suggest that the redox environment may influence the bioactivity of the bacterial metabolome/secretome. A recent study showed that in cancers, including breast cancer, ileopathy occurs, leading to slower intestinal passage [82]. Slower passage changes the composition of the microbiome and may also affect the redox balance in the GI tract [83,91]. 

Amino acid homeostasis is hampered in most breast cancer patients [92,93]. This is reflected by the metabolites identified as bioactive in this study, which are mainly involved in amino acid metabolism. Vanillic acid and trans-ferulic acid are derivatives of l-tyrosine. These metabolites are found in extracts of bacteria or plants tested for anticancer activity. Vanillic acid can act as a pro-oxidant and suppress cell proliferation [94]. 

Other compounds, such as hydrocinnamic acid, 3-hydroxyphenylacetic acid, and 4-hydroxyphenylacetic acid, contain an aromatic ring and a polar carboxylic acid moiety. Lactic-acid-producing bacteria produce hydrocinnamic acid [95]. The flavonoid compound, 3-hydroxyphenylacetic acid, is generated via the degradation of quercetin derivatives, which are synthesized by *Clostridiales* in the human microbiome [92,93,94,95,96,97,98,99]. The metabolite, 3-hydroxyphenylacetic acid, has cytoprotective features [100] and can bind to the γ-hydroxybutirate receptor [101,102]. The metabolite, 4-hydroxybenzoic acid, is synthesized from chorismic acid and can bind to the estrogen receptor [103], peroxisome-proliferator-activated receptor (PPAR)γ [104], and G-protein-coupled receptor 40 (GPR40) [104].

Interestingly, multiple metabolites identified in this study, including glycolic acid, hydrocinnamic acid, 2,3-butanediol, 3-hydroxyphenylacetic acid, and 4-hydroxyphenylacetic acid, have antibacterial activity or are involved in quorum sensing [99,103,105,106,107,108,109]. This implies that these metabolites modulate not only the mammary tumor cells but also the microbiome. 

Vanillic acid, 4-hydroxybenzoic acid, and 3-hydroxyphenylacetic acid supported proliferation at a specific concentration, i.e., their dose–response curve had an inverted V-shape. This behavior is similar to certain previously identified antineoplastic bacterial metabolites, including indoxyl-sulfate [32] and indol propionic acid [31] (in both cases, V-shape was observed). An inverted V-shape curve indicates the consecutive activation of a receptor-inducing proliferation at low concentrations followed by the activation of another receptor inhibiting proliferation at higher concentrations. Toxicity may also yield similar features at high concentrations; however, the compounds were used in the reference range, making this scenario unlikely.

## 4. Materials and Methods

### 4.1. Chemicals

All chemicals were purchased from Sigma (St. Louis, MO, USA) unless stated otherwise. The source of the bacterial metabolites is provided in Table 1. TGFβ was from Thermo Scientific (Waltham, MA, USA; Cat. No. #100.21), and SB-431542 was from Sigma (Cat. No. S4317).

### 4.2. Cell Culture

The 4T1 murine breast cancer cell line was maintained in RPMI-1640 (Sigma) medium, supplemented with 10% fetal bovine serum (FBS), 1% penicillin/streptomycin, 2 mM l-glutamine, and 1% pyruvate at 37 °C with 5% CO_2_. A2780 cells were cultured in RMPI 1640 medium supplemented with 10% FBS, 2 mM glutamine, and 1% penicillin-streptomycin. MCF-7 cells were maintained in Minimal Essential Medium (MEM, Sigma), 10% FBS (Sigma), 1% penicillin/streptomycin (Invitrogen, Waltham, MA, USA), and 2 mM l-Glutamine. Capan2 (human pancreatic adenocarcinoma cells) were maintained in MEM (Sigma; cat. no. M8042) containing 10% FBS, 1% penicillin-streptomycin, and 2 mM glutamine at 37 °C with 5% CO_2_.

### 4.3. Cytochemistry and Fluorescent Microscopy

Cells were grown on PerkinElmer CellCarrier Ultra 96-well (Waltham, MA, USA) plates until 70% confluency. Samples were fixed with 4% formaldehyde for 15 min, permeabilized with 1% Triton-X 100, and blocked with 1% BSA/PBS solution. Nuclei were labeled with DAPI (Thermo, Cat. No. R3706) for 5 min. Actin filaments were labeled with Texas Red-X phalloidin (Thermo, T7471) for 1 h. Fluorescent images of the nuclei were acquired at a resolution of 1080 × 1080 pixels (px) with a 10× objective (N.A. 0.3, 1.196 μm/px). DAPI signals (excitation/detection λ: 405/456) were detected in the whole well with 21 fields. Morphology images (i.e., fluorescent detection of phalloidin-stained cells) were acquired at 2160 × 2160 px image resolution, with a 20× objective (N.A 0.4, 0.299 μm/px). DAPI and Texas Red-X Phalloidin signals (excitation/detection λ: 561/599) were detected in 25 fields from each well. Both image sets were acquired with PerkinElmer Opera Phoenix High Content Screening System (Waltham, MA, USA).

### 4.4. Cell Morphology Analysis

The acquired images were segmented and analyzed using Harmony 4.8 software (PerkinElmer). The nuclei and the cytoplasm were segmented and textured, and the signal intensity and position of the segmented objects were calculated. Using the calculated morphological properties of TGFβ1-treated, SB-431542-treated, and control cells, an embedded linear regression model was trained to classify the cells into epithelial or mesenchymal morphology groups.

### 4.5. Training Dataset—DAPI

The training dataset was created from a database of DAPI-stained images from A2780 human ovarian cancer, 4T1 mouse breast cancer, Capan2 human pancreatic cancer, and MCF7 human breast cancer cell lines. Eighty-two images were selected as a training dataset from our image database to provide the highest possible variations in a single image and between images. Images were considered good examples if they had (1) relatively high or low fluorescent signals, (2) contained staining errors or artifacts, or (3) were over-confluent with nuclei of mixed nuclear morphology that covered a part of the well edge. Nuclei were segmented with CellProfiler (Broad Institute, Cambridge, MA, USA) followed by manual correction. The original dataset was augmented using the Albumentations (Python3) library, including 90° rotation, cropping, and brightness changes. The augmentation resulted in 277 pairs of gray-scale images and the corresponding segmented images. The order of the images in the augmented dataset was randomized and split into training, test, and validation sets at a ratio of 80:10:10. 

### 4.6. Training Parameters

Using this dataset, a VGG-UNet segmentation model was trained. The model’s architecture was extracted from the Keras-segmentation 0.3.0 library (Python3). The model was trained on a central processing unit for two epochs, eight images per batch through 33 steps. The model used for evaluating the validation dataset had a loss of 0.0512 and an accuracy of 0.9874. The code is shared at the link provided in the “Data Availability” section.

### 4.7. Nuclei Counting

Nuclei were segmented using Cellprofiler’s (https://cellprofiler.org/, version 3.1.8) integrated object segmentation method (adaptive Otsu method with three classes—middle class assigned to background). The objects were counted on each image. Parallel to this, the trained semantic segmentation model was applied as a substitute for CellProfiler’s segmentation method, followed by object counting with CellProfiler.

### 4.8. Sulforhodamine B Cell Proliferation Assay

The sulforhodamine B (SRB) assay was used to assess cell proliferation as described in [110,111]. The timing of the proliferation assay was set up as a function of the doubling time of the cell lines.

### 4.9. Western Blot

Western blotting was performed similar to [112,113]. Briefly, cells and tissues were lysed with RIPA buffer (50 mM Tris, 150 mM NaCl, 0.1% SDS, 1% Nonidet P-40, 1 mM Na_3_VO_4_, 1 mM NaF, 0.5% sodium deoxycholate, 1 mM phenylmethylsulfonyl fluoride, and protease inhibitor mixture, pH 8.0) and boiled in 50 mM Tris, 2% (*w*/*v*) SDS, 3.34% (*v*/*v*) glycerol, and 16.67 mM β-mercaptoethanol. Proteins were separated by 10% SDS-PAGE and transferred onto a nitrocellulose membrane (Bio-Rad Laboratories, Supported, Hercules, CA, USA). Antibodies used in this study were diluted in 5% BSA solution and are shown in Table 2. The secondary antibody was an anti-rabbit IgG HRP-linked antibody (Cell Signaling Technology, Danvers, MA, USA). Labeled proteins were detected using the Chemidoc Touch Imaging System with Supersignal West Pico and Supersignal West Femto ECL Kit (Thermo Fisher Scientific, Waltham, MA, USA). Blots were quantified using densitometry using Image Lab (Bio-Rad) software (version 6.1). All blots are shown in the Supplementary Materials. 

### 4.10. Statistics

All graphs and statistical analyses were generated using GraphPad Prism v.8.0.1 software. Normality was assessed using the Shapiro–Wilk test. If the dataset was normally distributed or normally distributed after log transformation, one-way ANOVA was applied. If normal distribution was not achieved, the Kruskal–Wallis test was performed. The statistical tests can be accessed in the primary data files. The dose–response curve of the metabolites was subjected to statistical analysis separately for each metabolite. P-values are indicated in the figure captions.

## 5. Conclusions

In this study, we identified bacterial metabolites that can modulate the behavior of breast cancer cells, similar to hormones. We screened a library of bacterial metabolites and identified nine bioactive metabolites (butyric acid, vanillic acid, glycolic acid, d-mannitol, trans-ferulic acid, 2,3-butanediol, 4-hydroxybenzoic acid, hydrocinnamic acid, and 3-hydroxyphenylacetic acid). The 4T1 murine breast cancer cells were treated with metabolites at concentrations corresponding to the reference serum concentrations of the metabolites. This step is very important to avoid toxicity or off-target effects at supraphysiological concentrations. However, yric acid, glycolic acid, d-mannitol, 2,3-butanediol, and trans-ferulic acid exerted cytostatic effects, while 3-hydroxyphenylacetic acid, 4-hydroxybenzoic acid, and vanillic acid exerted hyperproliferative effects on 4T1 cells. Furthermore, 3-hydroxyphenylacetic acid, 4-hydroxybenzoic acid, 2,3-butanediol, and hydrocinnamic acid inhibited epithelial-to-mesenchymal transition. We showed that the local redox environment may affect the bioactivity of the metabolites; hence, the local redox potential may affect the bacterial secretome and, consequently, the behavior of breast cancer cells. Out of the nine bioactive metabolites, 2,3-butanediol was the only one with cytostatic and anti-EMT properties. This study suggests that a large portion of the bacterially synthesized metabolites possess bioactivity, and these metabolites can modulate the behavior of cancer cells.

## Figures and Tables

**Table 1:** The specifics of the metabolites introduced to the metabolite library.

	Metabolite Name	Solvent	Catalog Number	Serum Reference Concentrations (µM)	Applied Concentrations (μM)
1	1-Butanol	PBS	Sigma B7906-500ML	0–0.27 [52]	0.005, 0.015, 0.044, 0.13, 0.4
2	1-Propanol	PBS	Sigma 96566-5ML-F	0–0.8 [52]	0.05, 0.1, 0.2, 0.4, 0.8
3	2,3-butanediol	PBS	Sigma B84904	0.5–0.9 [53]	0.48, 0.56, 0.66, 0.77, 0.9
4	3,4-dihydroxyphenyl acetic acid	PBS	Sigma 850217-14	0.0102–0.104 [54,55]	0.01, 0.018, 0.032, 0.058, 0.104
5	3-Hydroxyphenylacetic acid	PBS	Sigma H49901	0.11–0.174 [54]	0.106, 0.120, 0.136, 0.154, 0.174
6	3-Hydroxypropionic acid	PBS	Sigma 792659-1G	3, 6, 8 (individual values) [56]	0.5, 1, 2, 4, 8
7	4-aminobenzoic acid	EtOH	Sigma A9878-5G	5.01–32.0 [57]	0.3, 1.2, 3.6, 10.8, 32.4
8	4-hydroxybenzoic acid	PBS	Sigma 8218140250	0.019–0.035 [54]	0.019, 0.022, 0.026, 0.03, 0.035
9	4-Hydroxyphenylacetic acid	PBS	Sigma H50004-54	0.283–0.61 [54]	0.28, 0.36, 0.41, 0.5, 0.61
10	Acetic acid	PBS	VWR UN2789	23–254.4 [52,58,59,60,61]	15, 30, 60, 120, 240
11	Allantoin	DMSO	Sigma 05670	1.0–24.0 [62,63,64]	0.99, 2.2, 4.9, 10.8, 24
12	Butyric acid	PBS	Sigma B103500-5ML	1.39–14.15 [59,60,61]	1, 2, 4, 8, 16
13	d-alanine	PBS	Sigma A7377-5G	0–0.77 [65]	0.048, 0.96, 0.193, 0.385, 0.77
14	d-glutamic acid	PBS	Sigma G1001-1G	7.42–14.6 [65]	7.28, 8.66, 10.31, 12.27, 14.6
15	d-mannitol	PBS	Sigma M4125-10MG	no report, same concentrations as for d-mannose	6.25, 12.5, 25, 50, 100
16	d-mannose	DMSO	no data	13–73.87 [66,67,68]	6.25, 12.5, 25, 50, 100
17	Ethylene glycol	PBS	no data	no data, toxic (1.56 mg/kg)	1, 3, 9,27, 81
18	Formic acid	PBS	Sigma F0507-500ML	11.84–224.5 [69]	10, 30, 90, 270, 810
19	Glycolic acid	PBS	Sigma 124737	6.1–69 [56,70]	1, 3, 9,27, 81
20	Hippuric acid	PBS	Sigma 112003	1.5–21.2 [54,71]	0.024, 0.12, 0.6, 3, 15
21	Hydrocinnamic acid	EtOH	Sigma 135232-5G	0.131–0.354 [72]	0.128, 0.165, 0.213, 0.274, 0.354
22	Isobutyric acid	PBS	Sigma I1754-100ML	1.02–14.15 [59,61]	1.02, 1.97, 3.80, 7.33, 14.15
23	l-pipecolic acid	PBS	Sigma P2519	1.2–3.72 [73]	0.25, 0.5, 1, 2, 4
24	oxalic acid	PBS	Sigma 75688	6.5–35.5 [74,75]	6.5, 9.9, 15.2, 23.2, 35.5
25	Propionic acid	PBS	Sigma P1386-1L	4.86–15.33 [59,60,61]	1.25, 2.5, 5, 10, 20
26	Shikimic acid	DMSO	Sigma S5375-10MG	0.03–0.23 [76]	0.01, 0.03, 0.09, 0.27, 0.81
27	trans-ferulic acid	DMSO	Sigma 52229	0.04–15.7 [54,71]	0.016, 0.08, 0.4, 2, 10
28	Trimethylamine (TMA)	PBS	Sigma 92260	0.3–14.44 [77,78]	0.3, 0.79, 2.1, 5.4, 14.35
29	Trimethylamine-*N*-oxide (TMAO)	PBS	Sigma 317594	1.21–21.1 [79,80,81]	1.22, 2.48, 5.065, 10.33, 21.1
30	Vanillic acid	PBS	Sigma H36001	0.01–0.338 [54,71]	0.01, 0.024, 0.058, 0.140,0.338

**Table 2:** The antibodies used in the study.

Antibody	Catalog Number	Company	Dilution
SnaiI	3879S	Cell Signaling Technology	1:1000
Vimentin	5741S		
β-actin	A3854	Sigma	1:20,000

## Data Availability

Primary biological data for this manuscript and the code are available at https://figshare.com/s/8aa8f400a4c73de1d9dd (DOI: 10.6084/m9.figshare.21394695). Primary images should be solicited from the last author.

## Supplementary Materials

The following supporting information can be downloaded at: https://www.mdpi.com/article/10.3390/molecules28155898/s1.
