# Supplementary material for: Identification of Bacterial Metabolites Modulating Breast Cancer Cell Proliferation and Epithelial-Mesenchymal Transition
Source: Molecules. 2023 Aug 5;28(15):5898. doi: 10.3390/molecules28155898 (PMC10420980; doi:10.3390/molecules28155898)
Supplement: Supplementary file 1 [file molecules-28-05898-s001.zip › molecules-2507689-supplementary.pdf]

POC0000181 positive/negative CTL

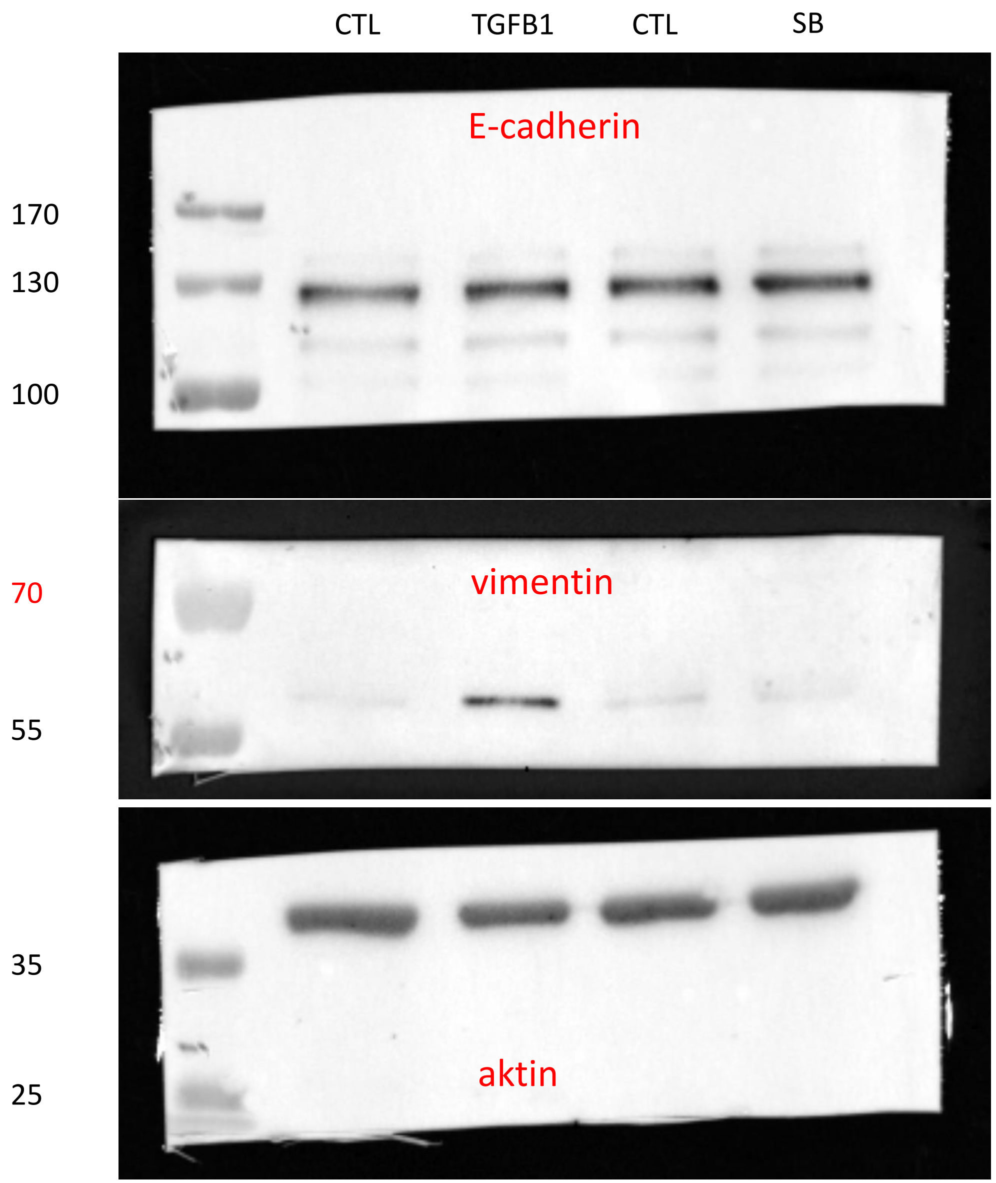

POC0000181 positive/negative CTL

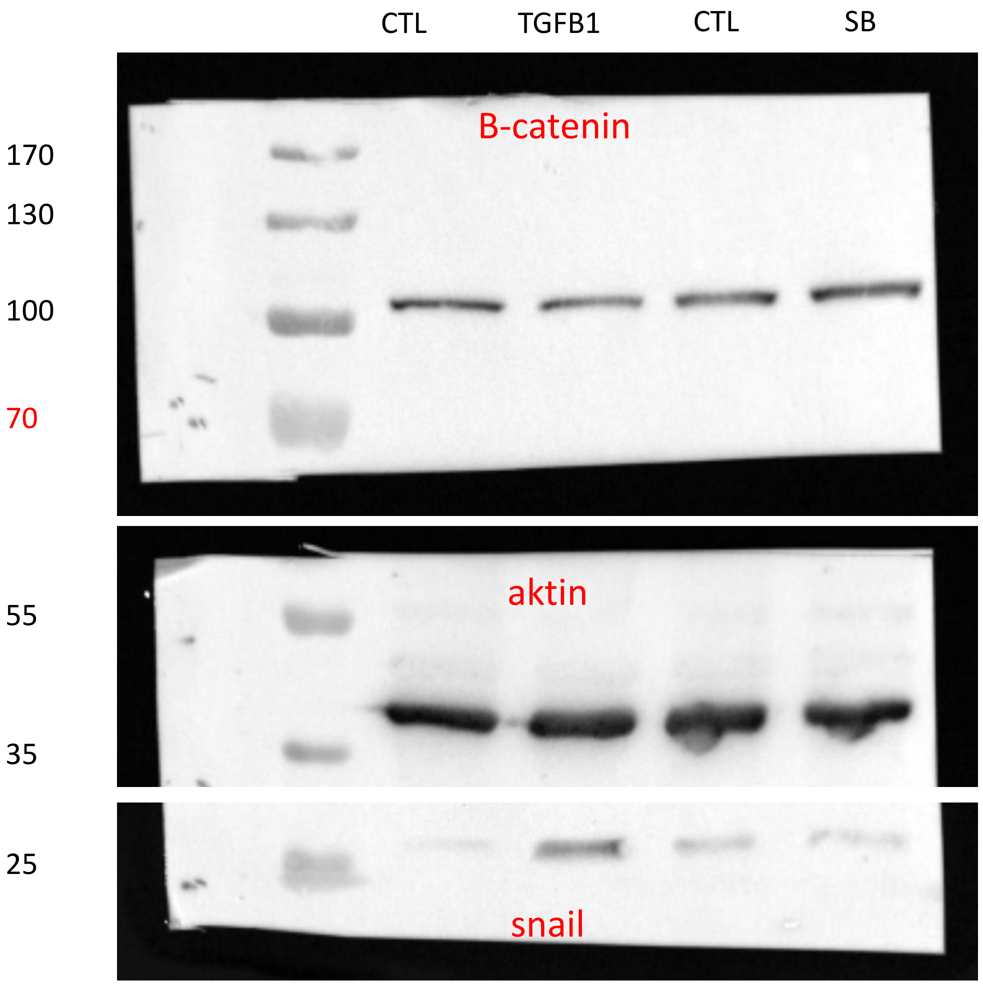

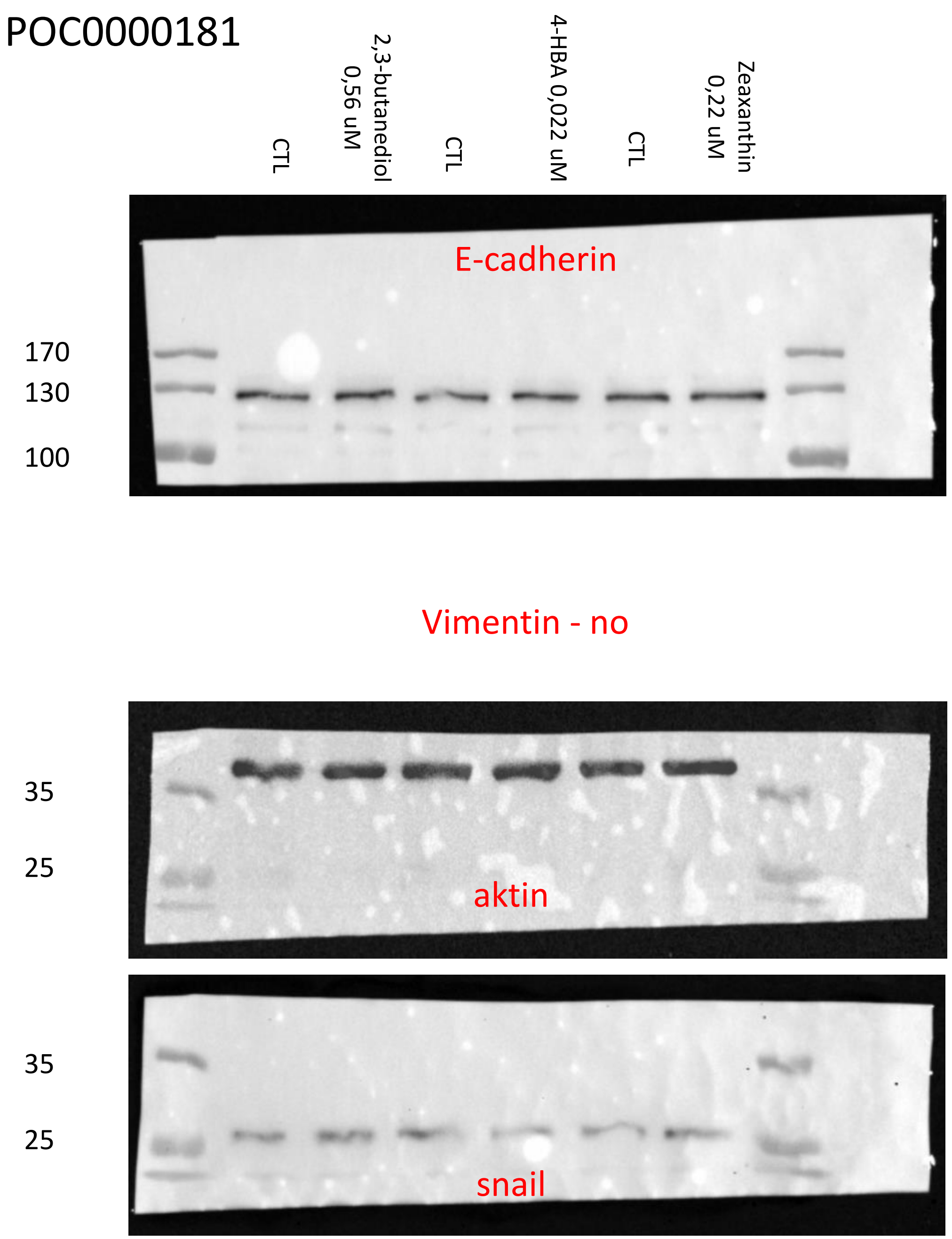
POC0000181
CTL
2,3-butanediol 0,56 uM
CTL
4-HBA 0,022 uM
CTL
Zeaxanthin 0,22 uM
E-cadherin
170
130
100
Vimentin - no
35
25
aktin
35
25
snail

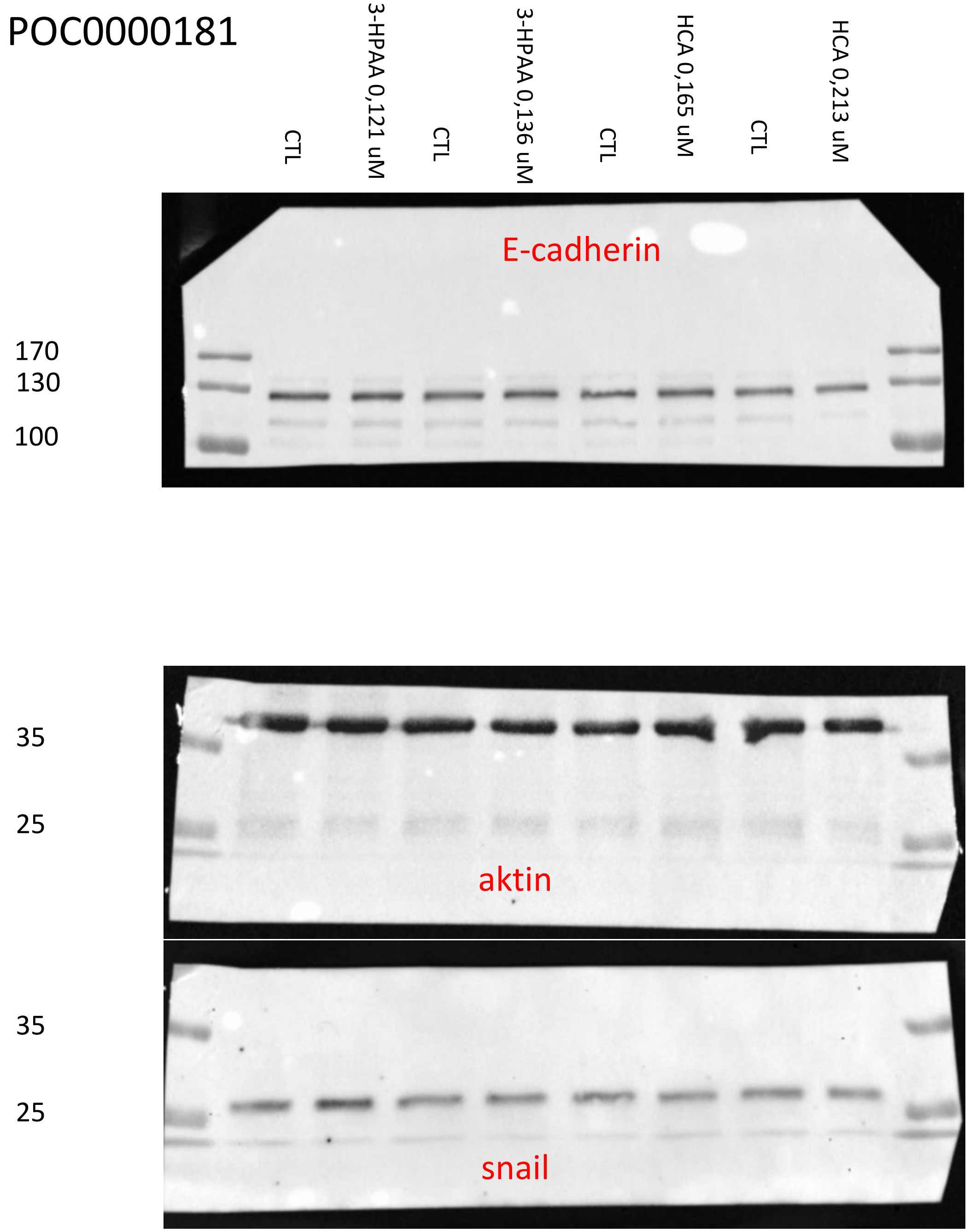
POC0000181
CTL
3-HPAA 0,121 uM
CTL
3-HPAA 0,136 uM
CTL
HCA 0,165 uM
CTL
HCA 0,213 uM
E-cadherin
170
130
100
35
25
aktin
35
25
snail

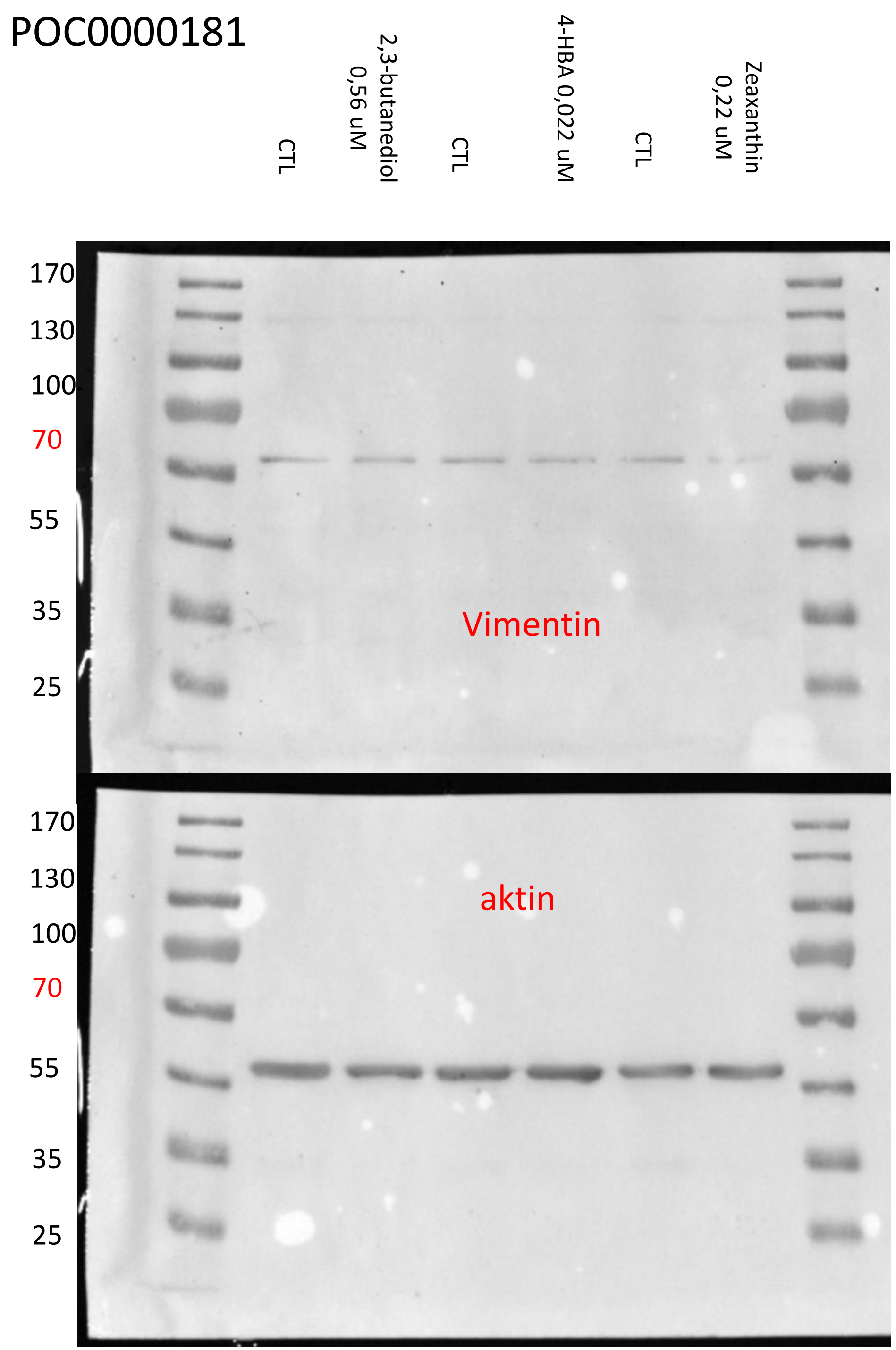
POC0000181
CTL
2,3-butanediol 0,56 uM
CTL
4-HBA 0,022 uM
CTL
Zeaxanthin 0,22 uM
170
130
100
70
55
35
25
Vimentin
aktin

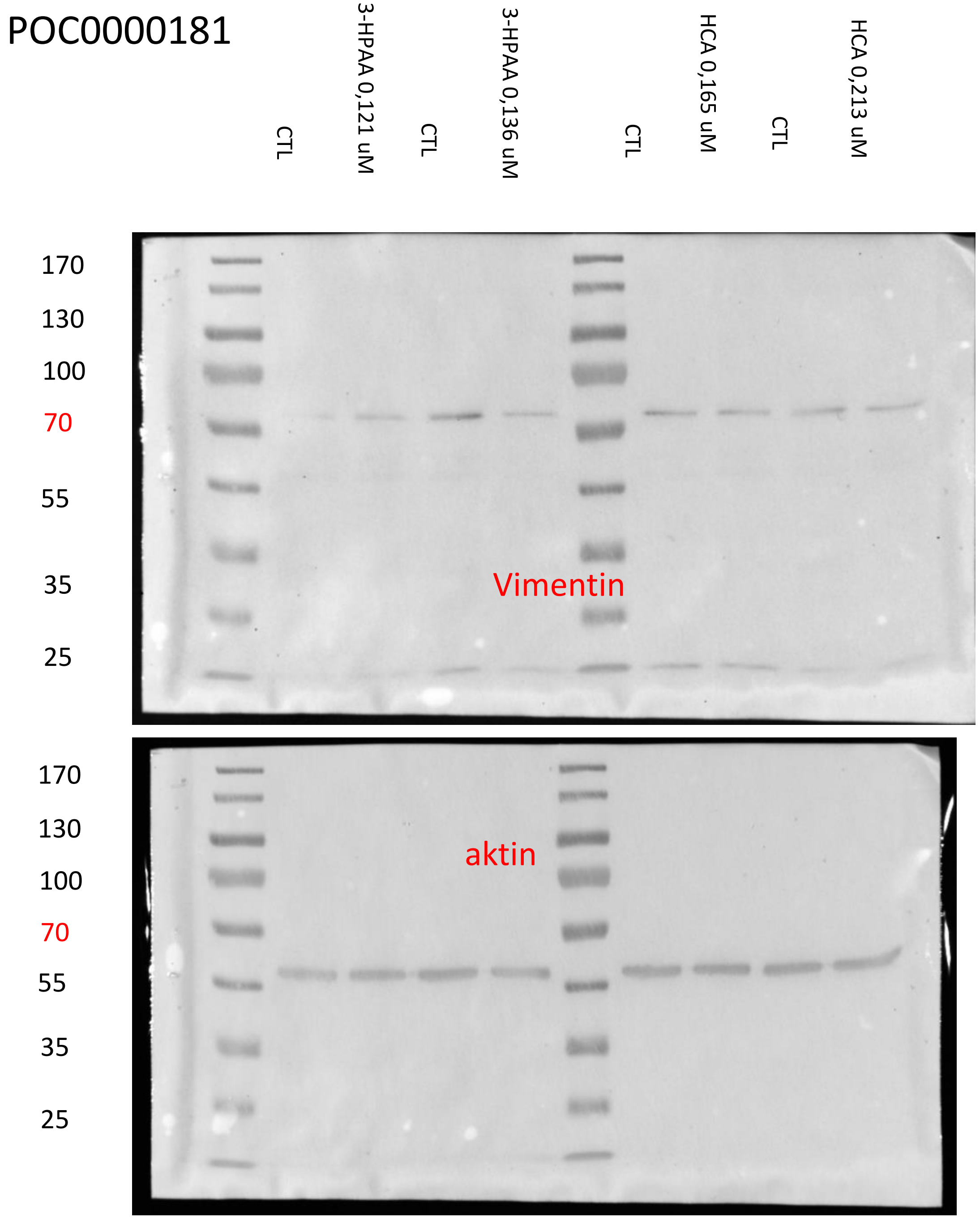
POC0000181
CTL
3-HPAA 0,121 uM
CTL
3-HPAA 0,136 uM
CTL
HCA 0,165 uM
CTL
HCA 0,213 uM
170
130
100
70
55
35
25
Vimentin
aktin

POC0000182 positive/negative CTL

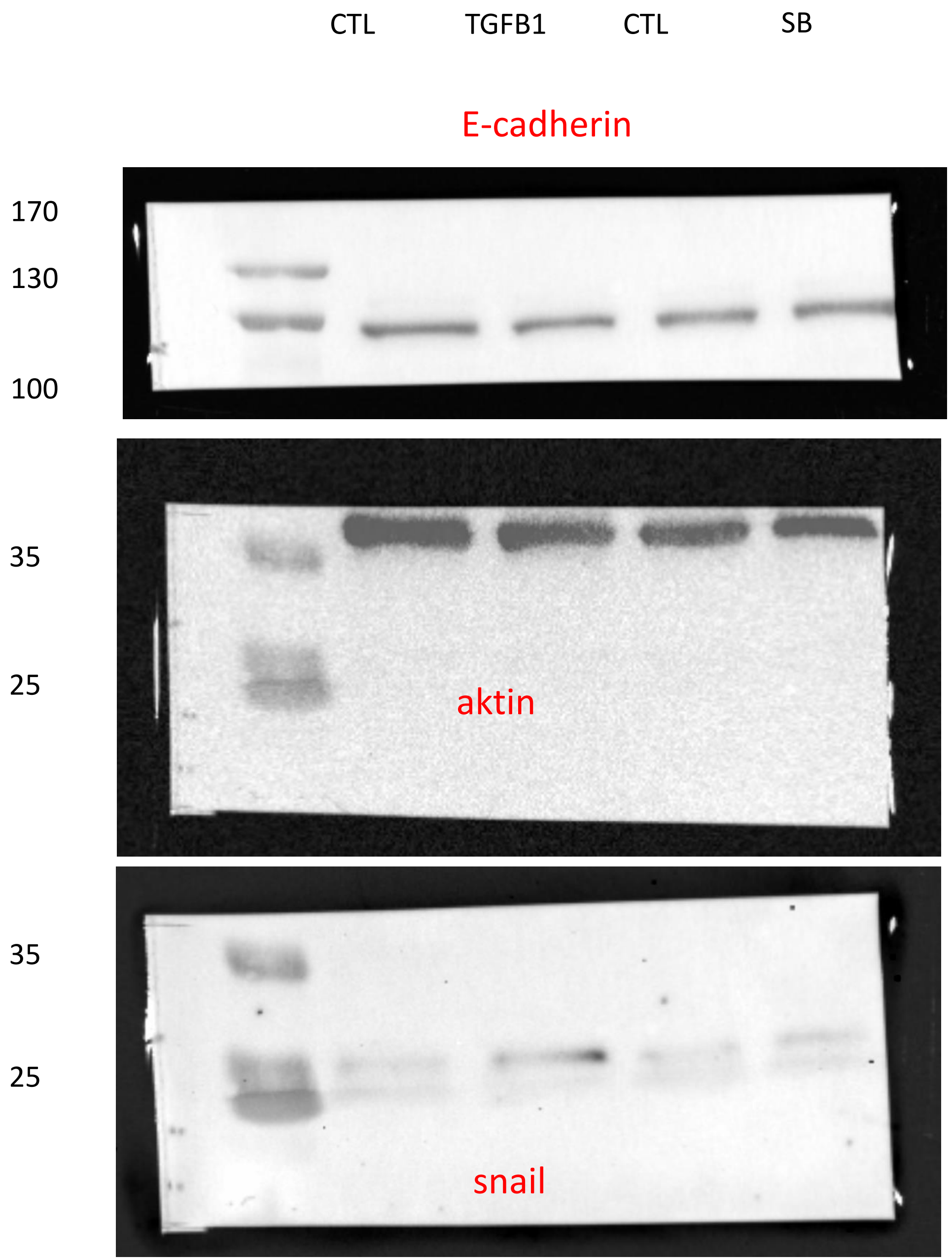

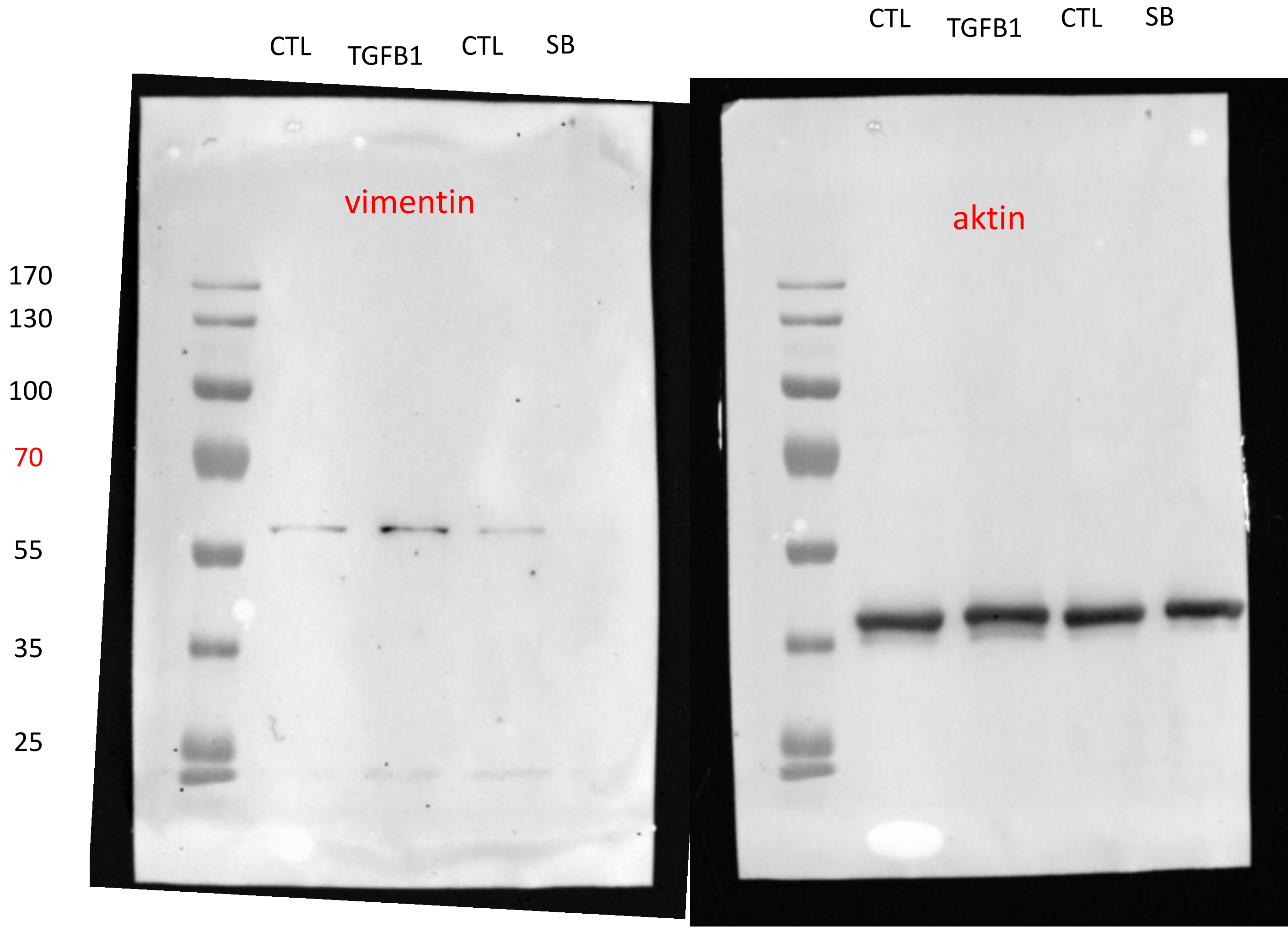

POC0000182

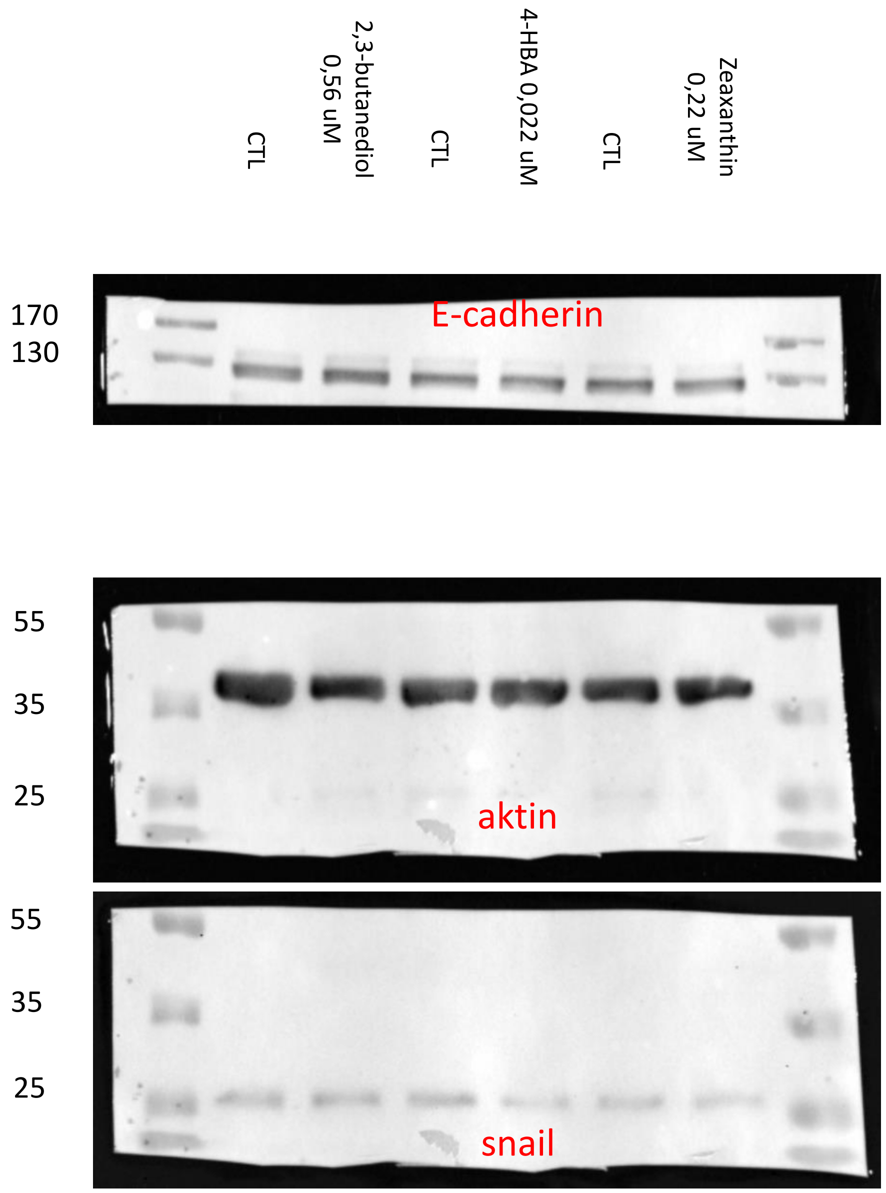

POC0000182

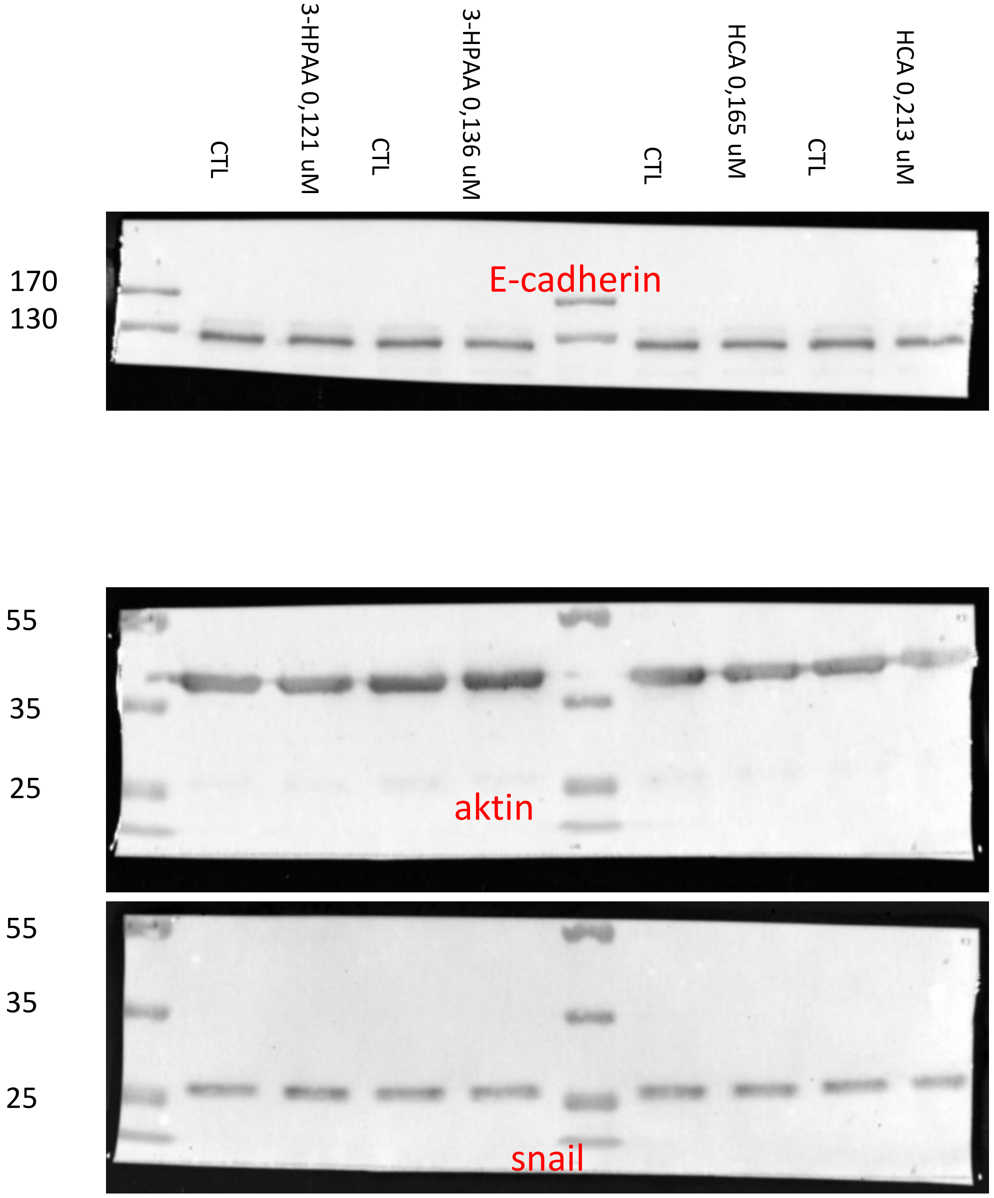

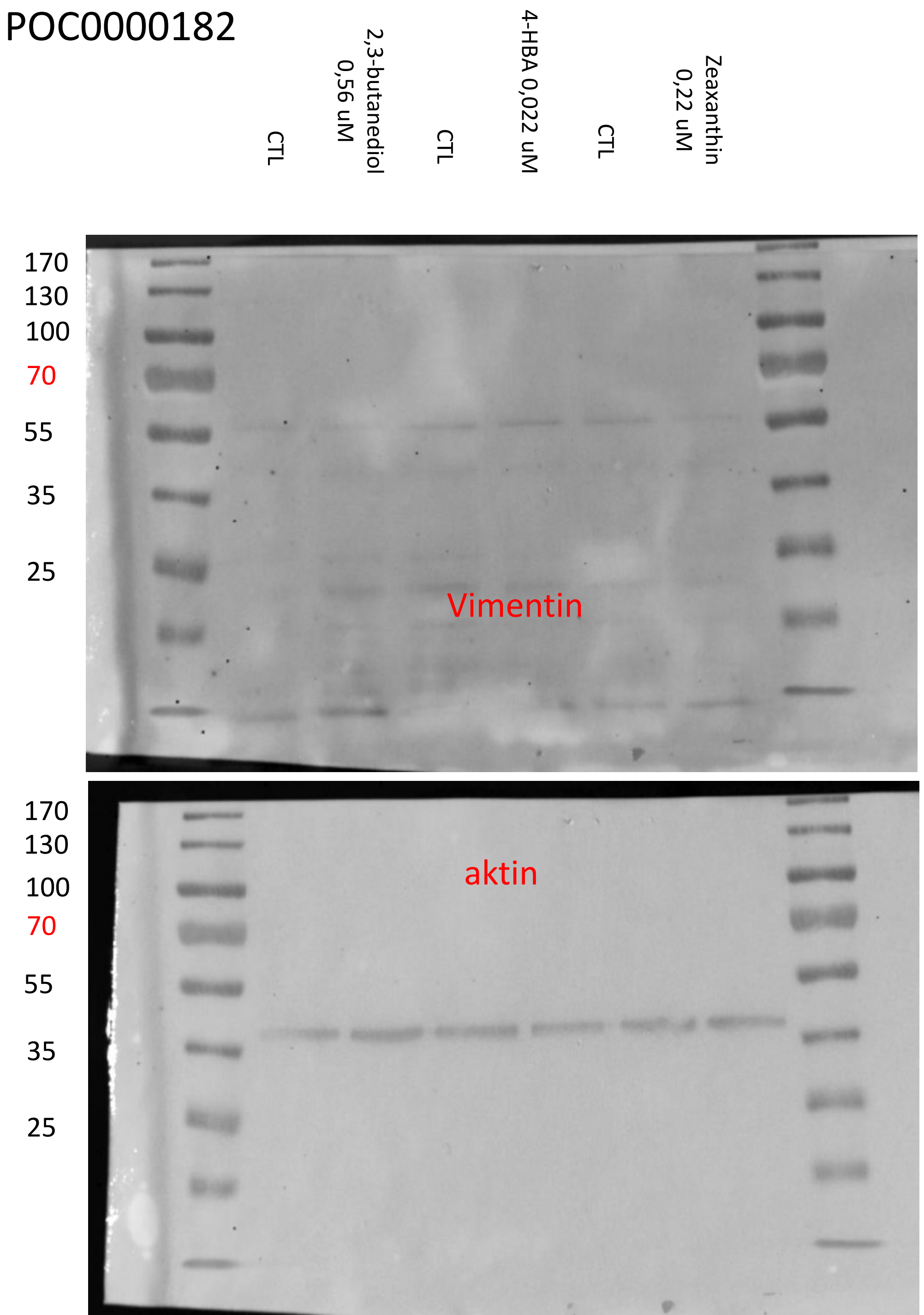
POC0000182
CTL
2,3-butanediol 0,56 uM
CTL
4-HBA 0,022 uM
CTL
Zeaxanthin 0,22 uM
170
130
100
70
55
35
25
Vimentin
170
130
100
70
55
35
25
aktin

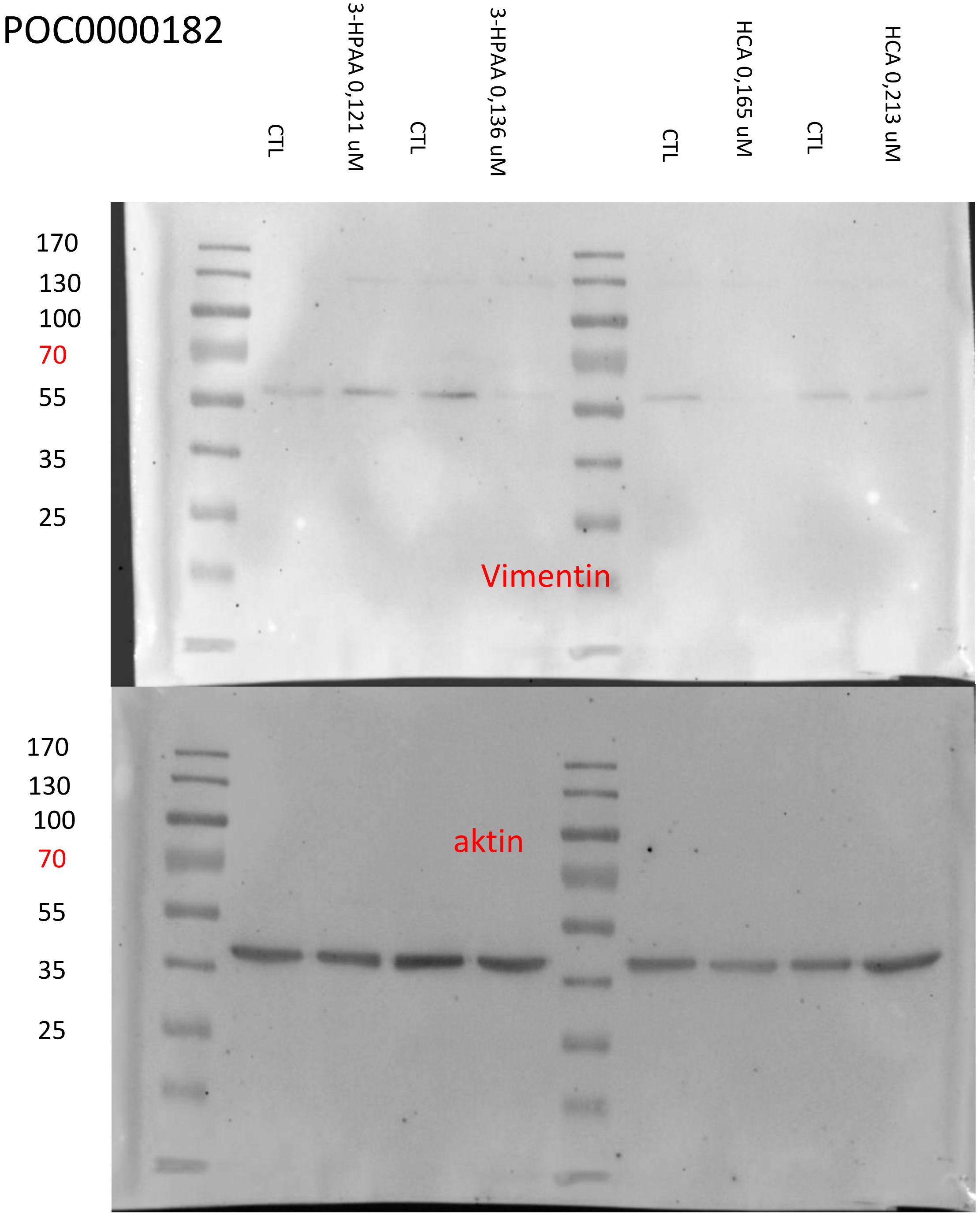
POC0000182
CTL
3-HPAA 0,121 uM
CTL
3-HPAA 0,136 uM
CTL
HCA 0,165 uM
CTL
HCA 0,213 uM
170
130
100
70
55
35
25
Vimentin
170
130
100
70
55
35
25
aktin

POC0000183 positive/negative CTL

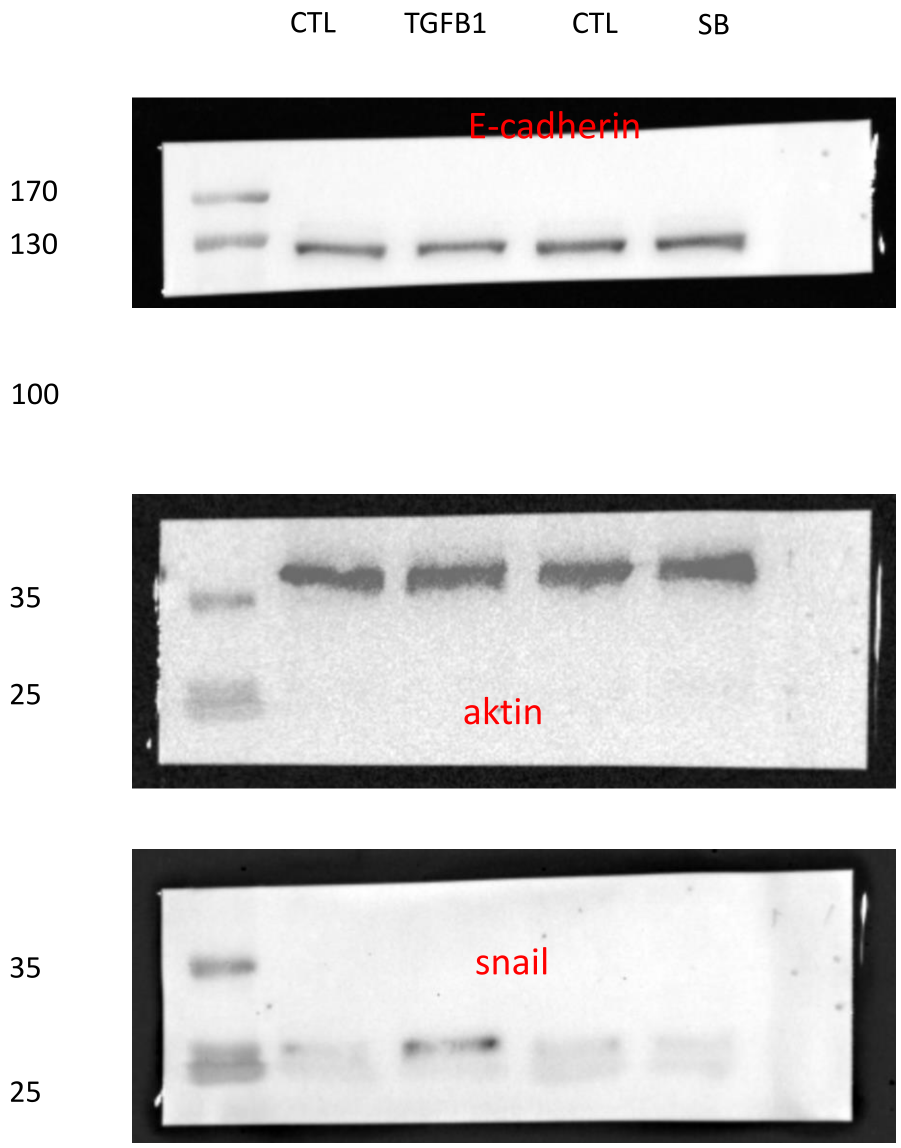

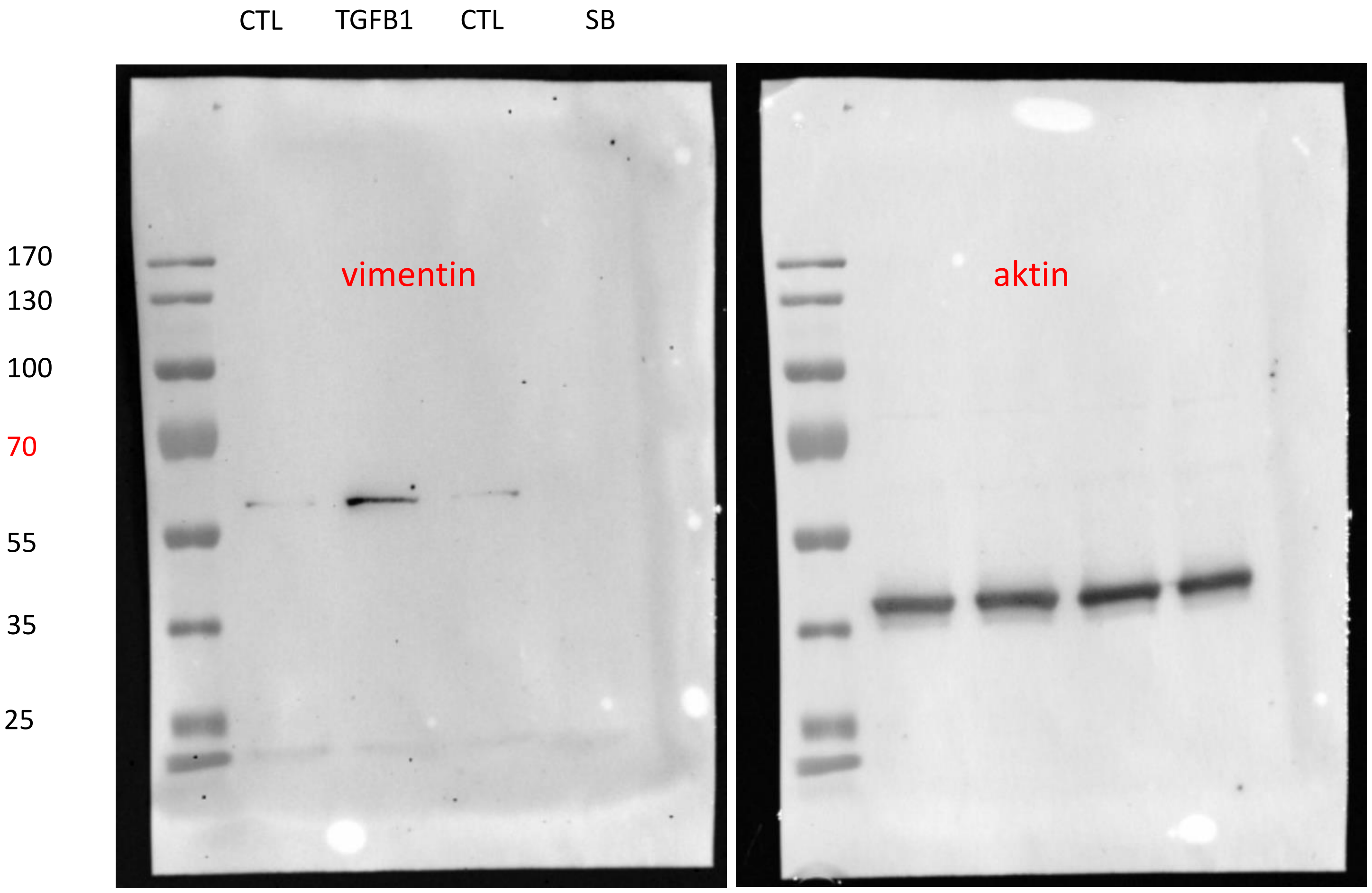

POC0000183 I.

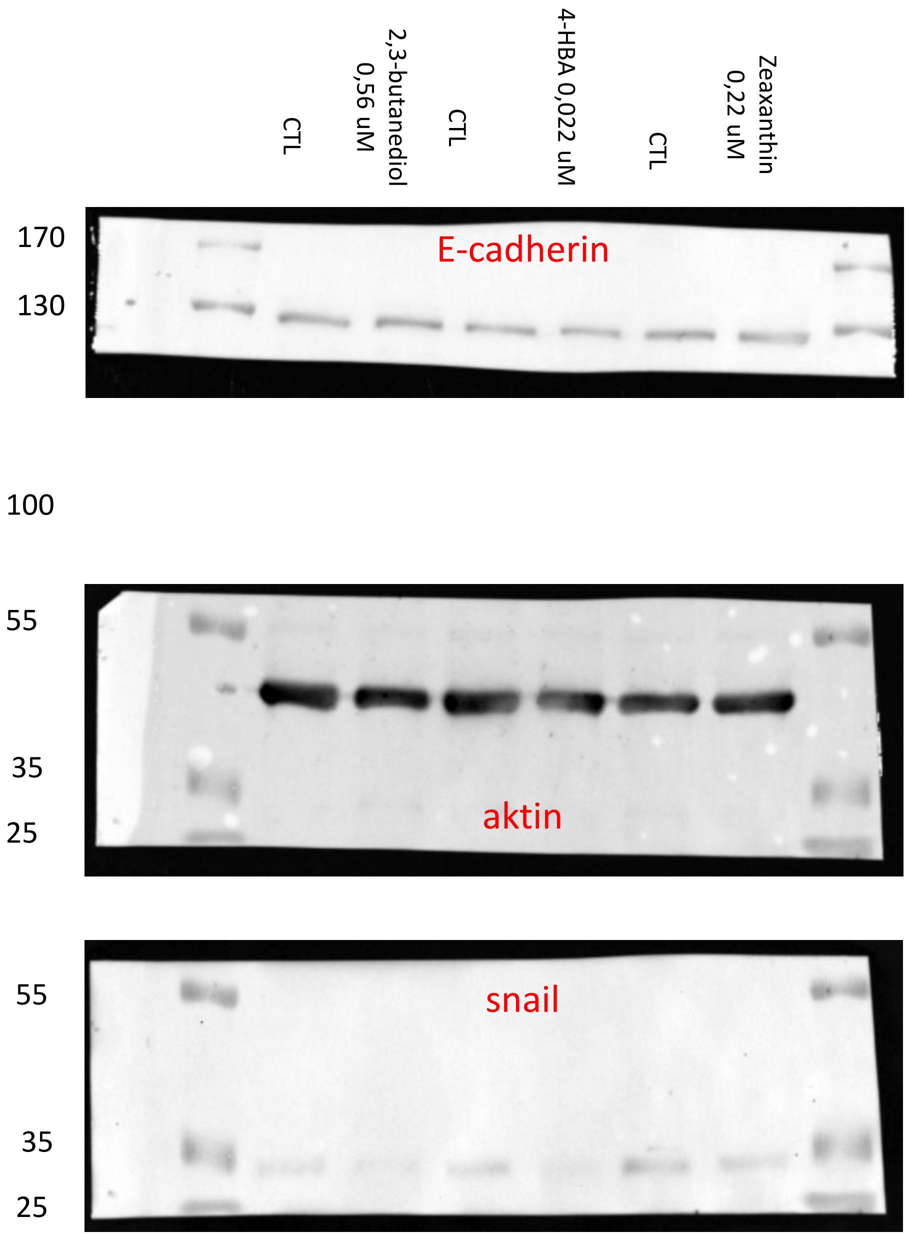

POC0000183 I.

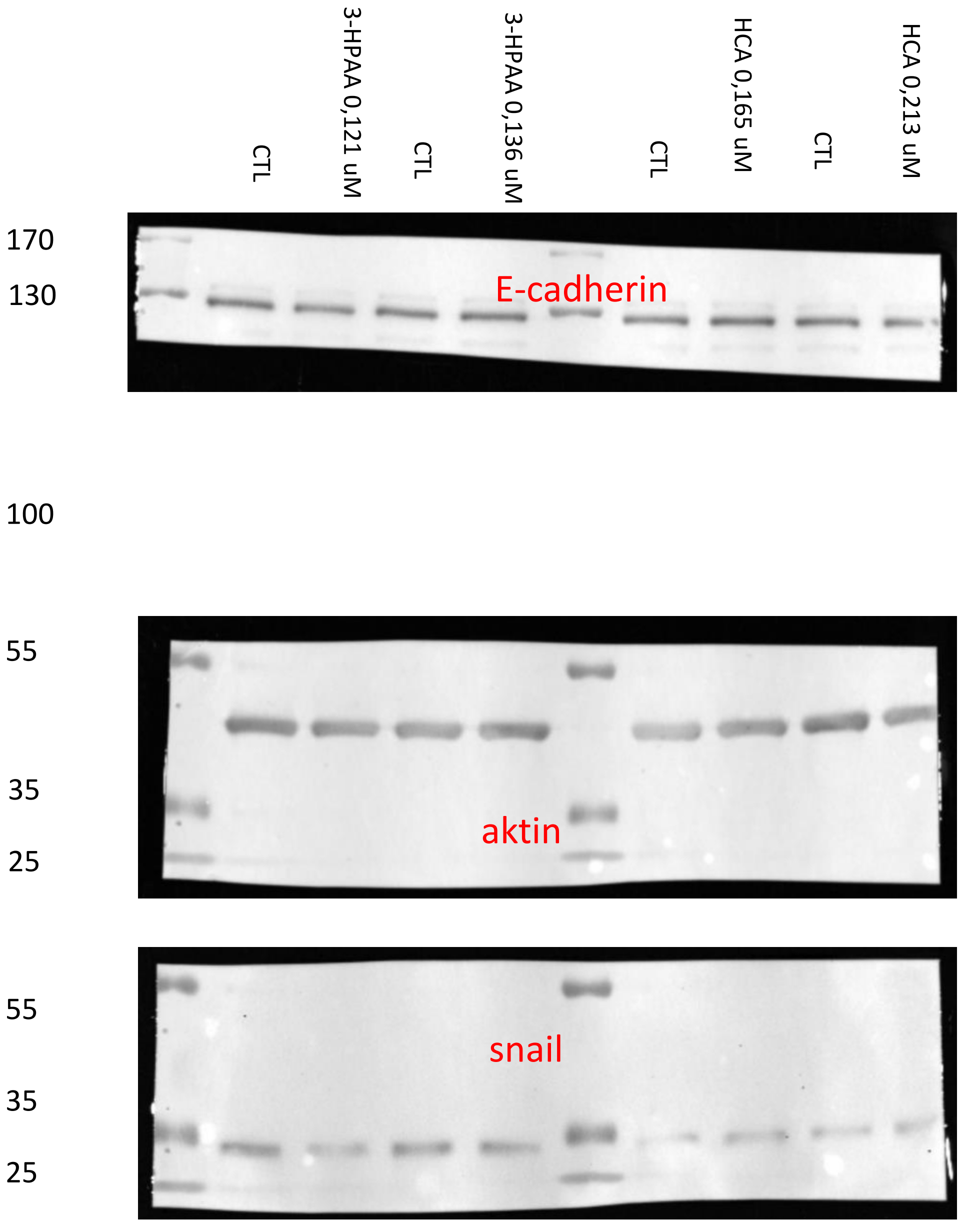

POC0000183 I.

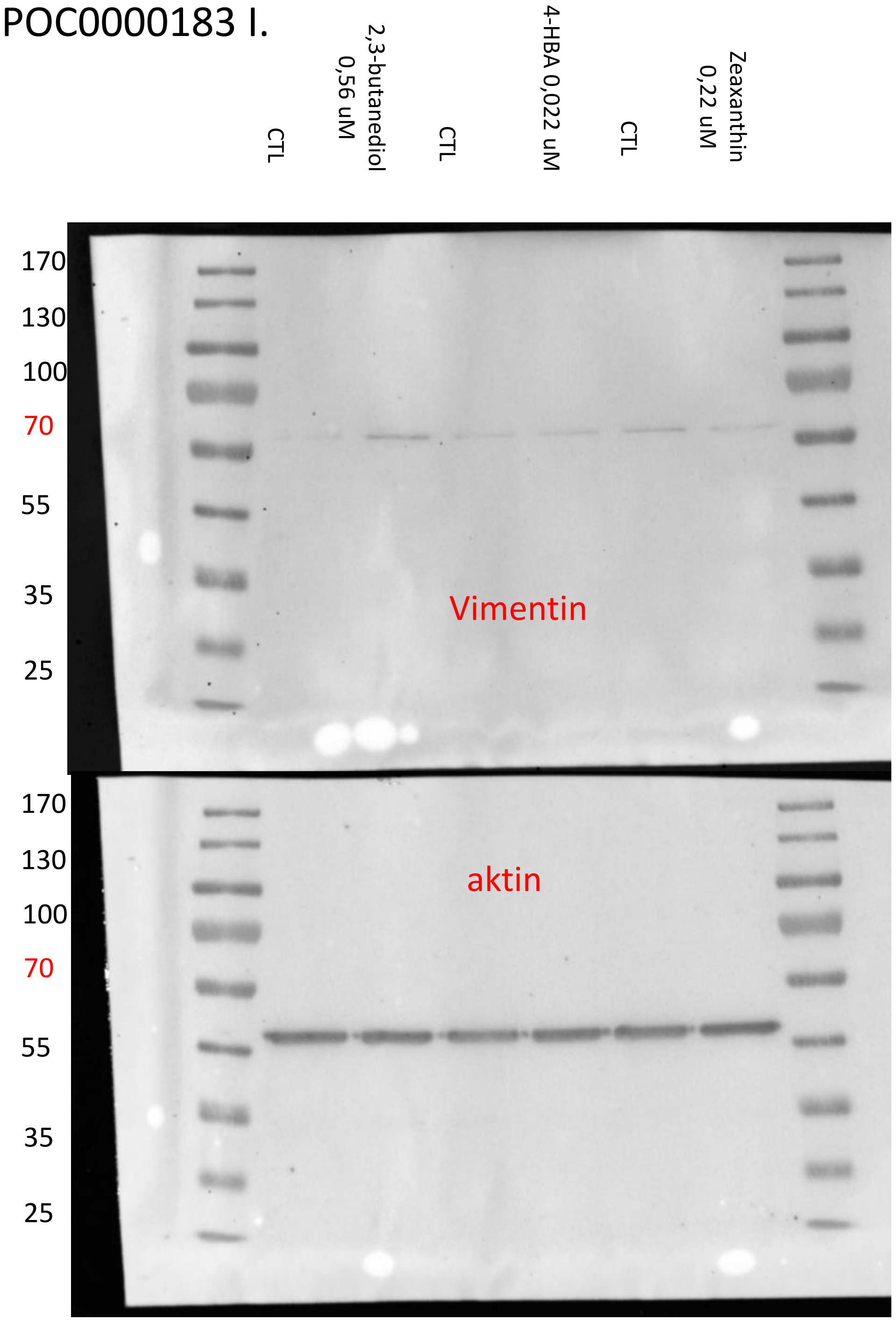

POC0000183 I.

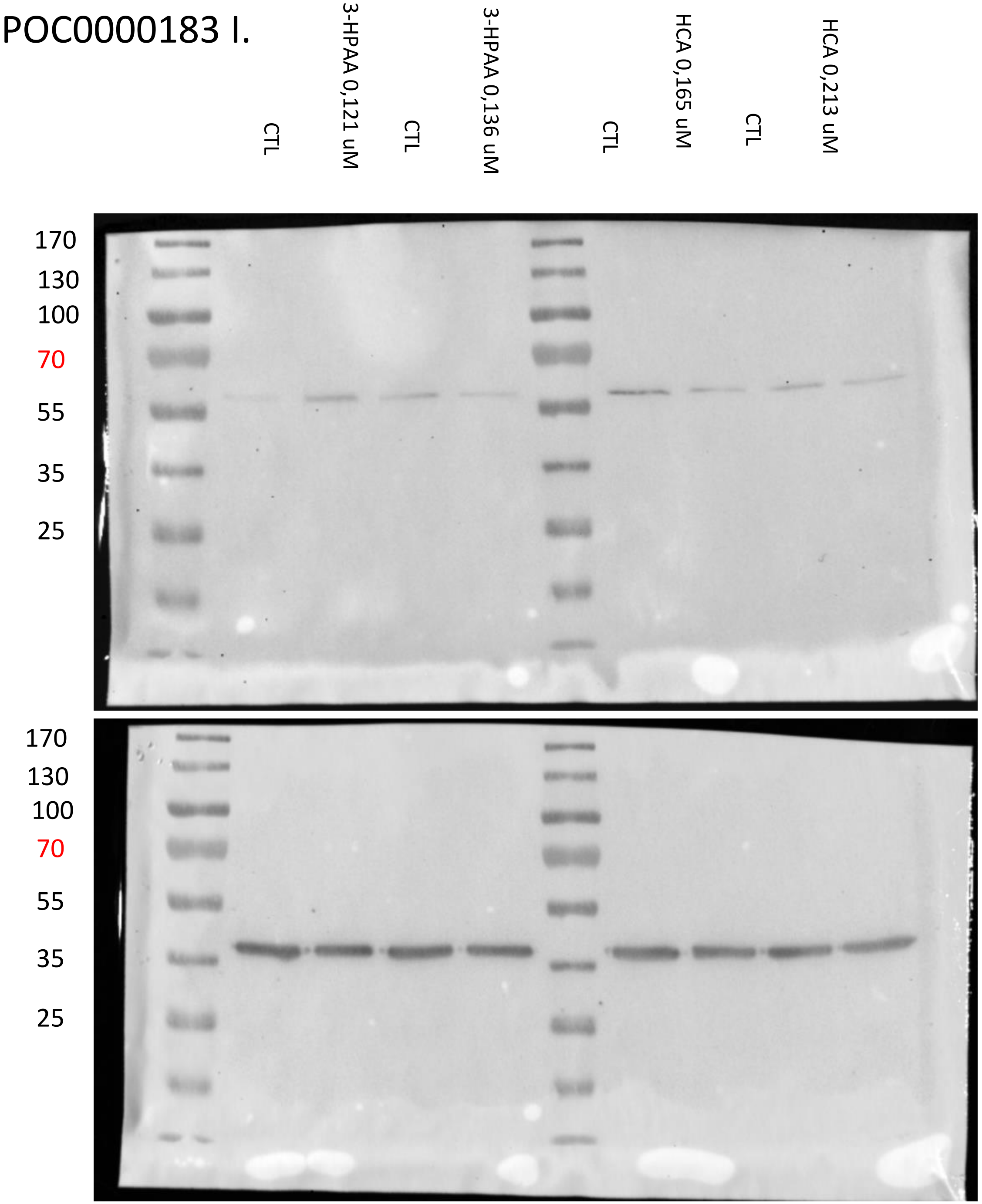

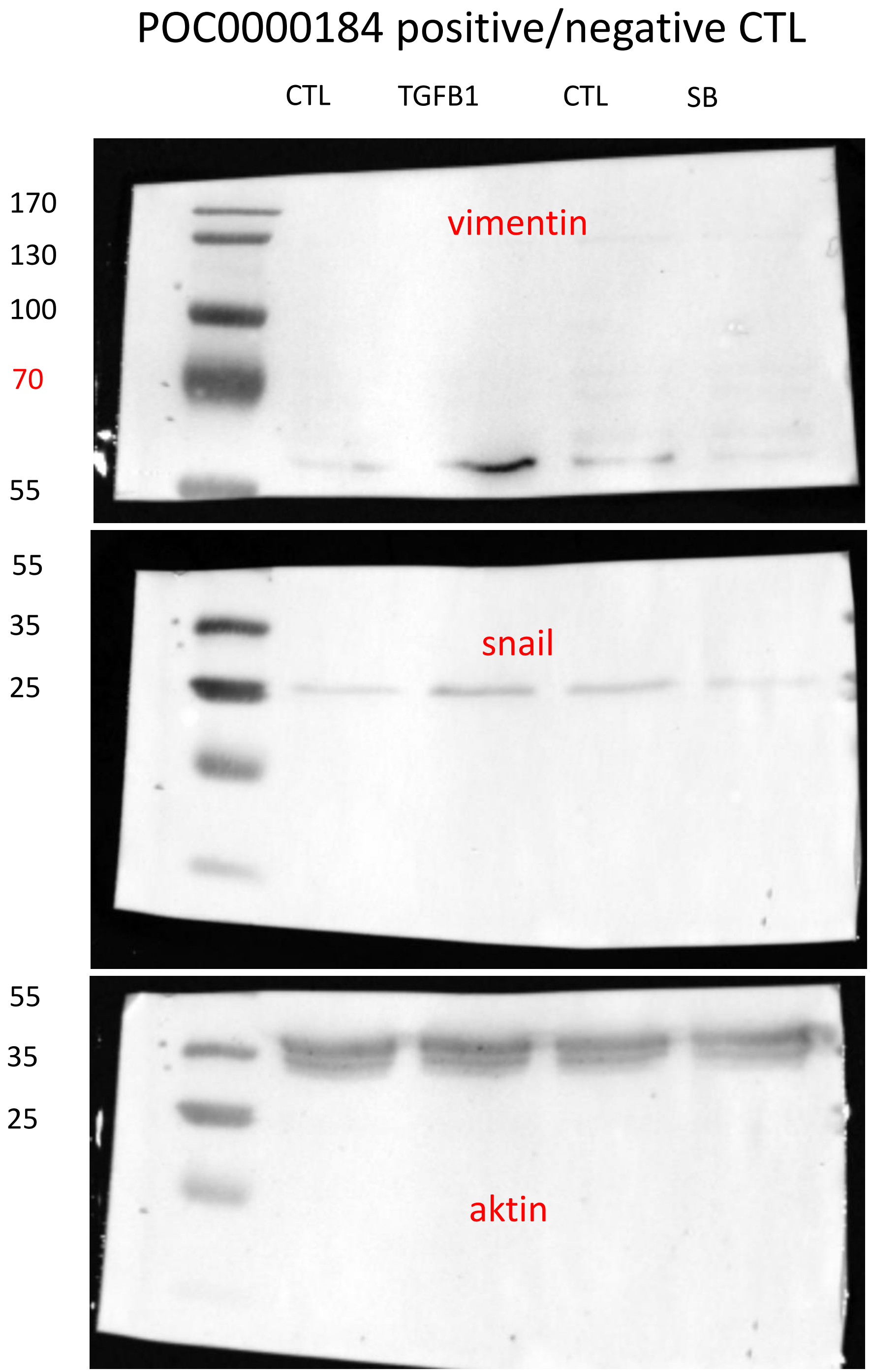
POC0000184 positive/negative CTL
CTL
TGFB1
CTL
SB
170
130
100
70
55
vimentin
55
35
25
snail
55
35
25
aktin

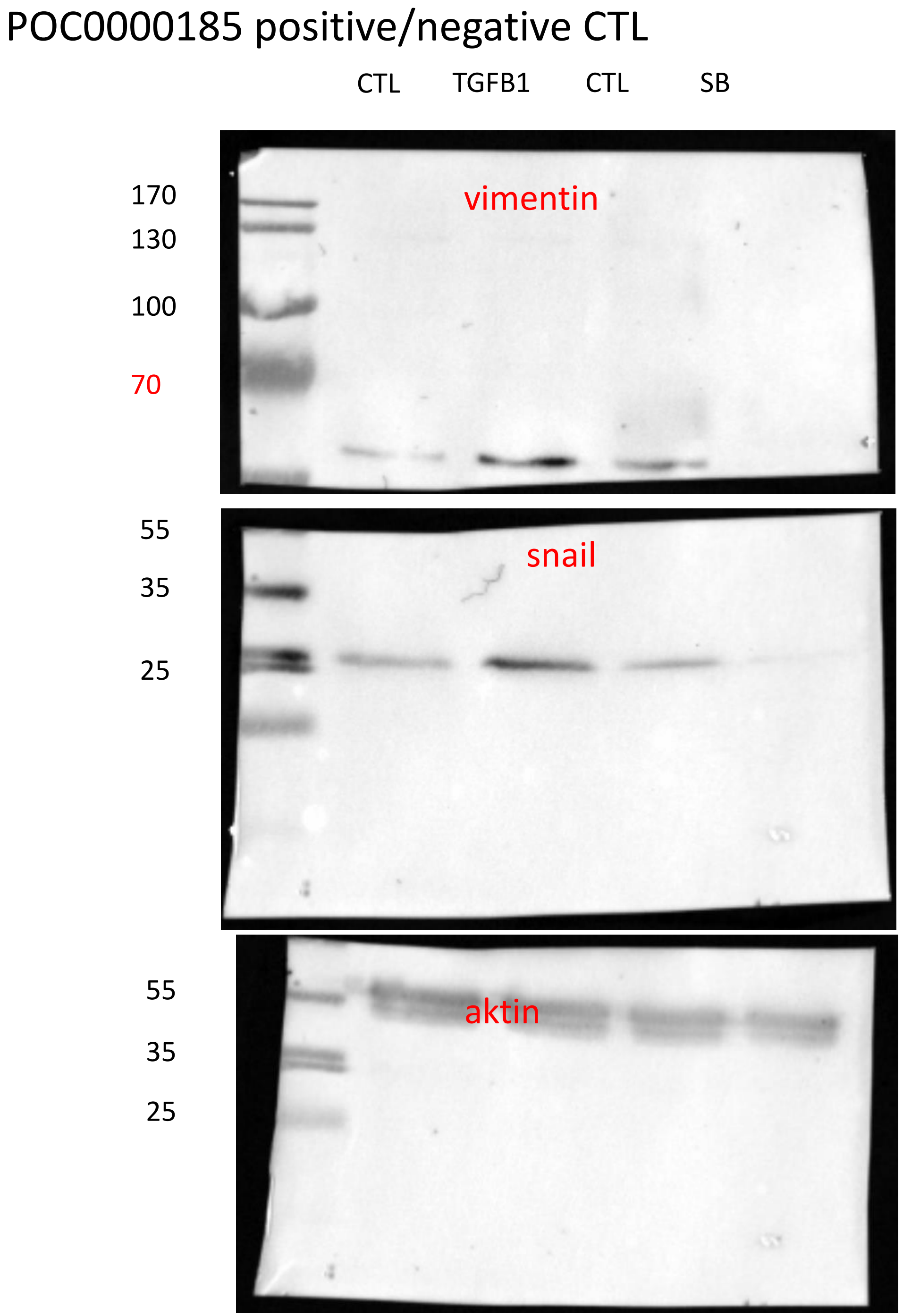
POC0000185 positive/negative CTL
CTL
TGFB1
CTL
SB
170
130
100
70
vimentin
55
35
25
snail
55
35
25
aktin

POC0000184

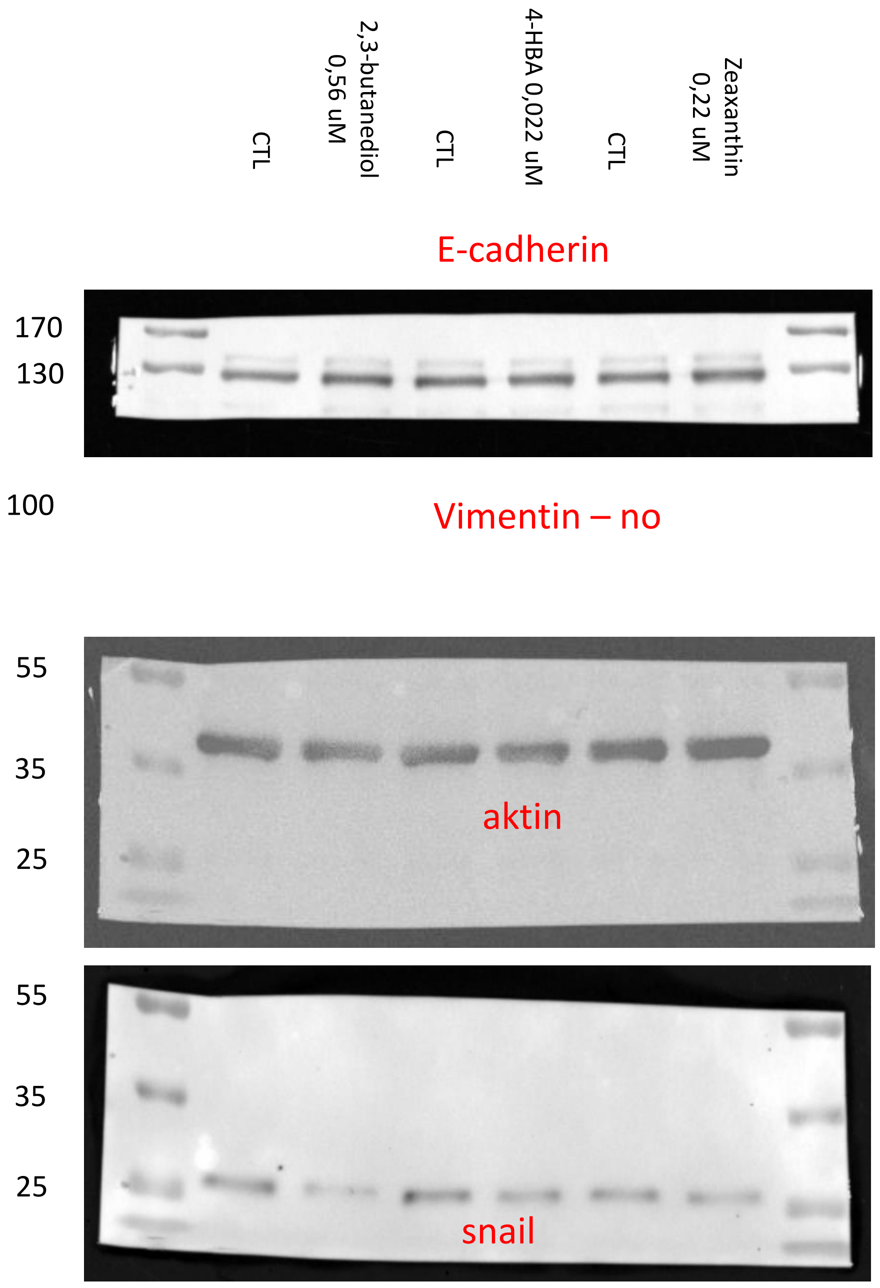

POC0000184

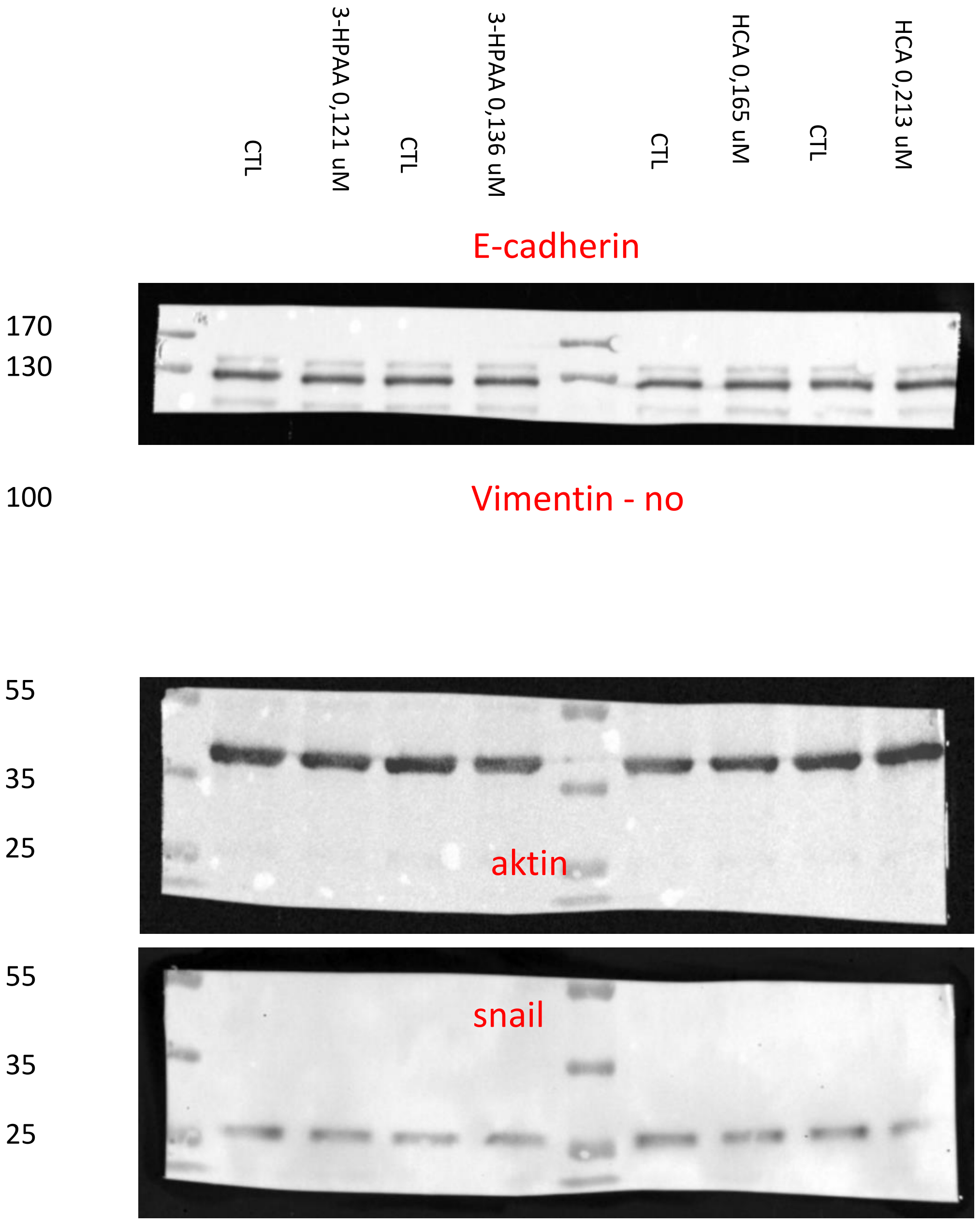

POC0000184

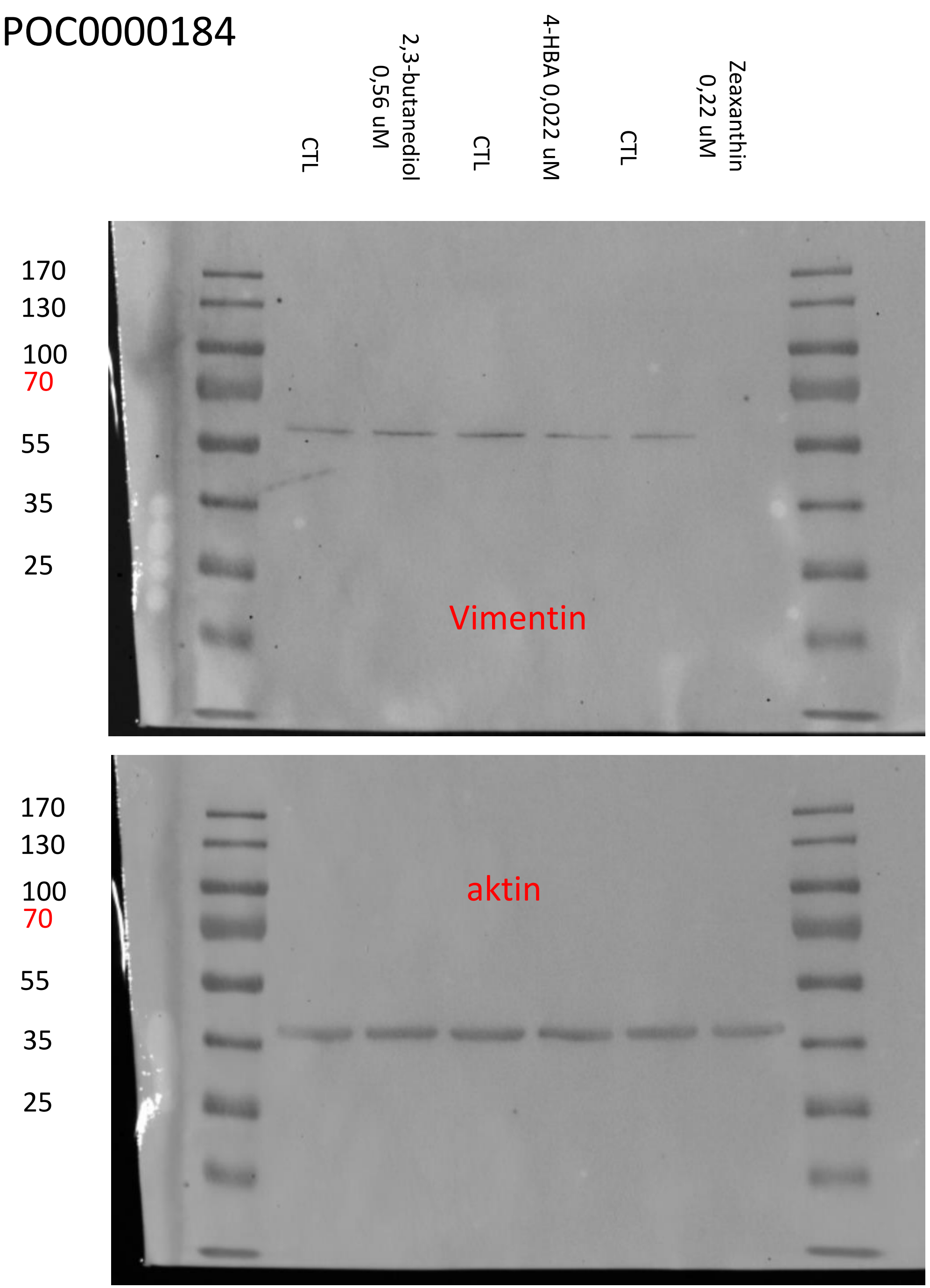

POC0000184

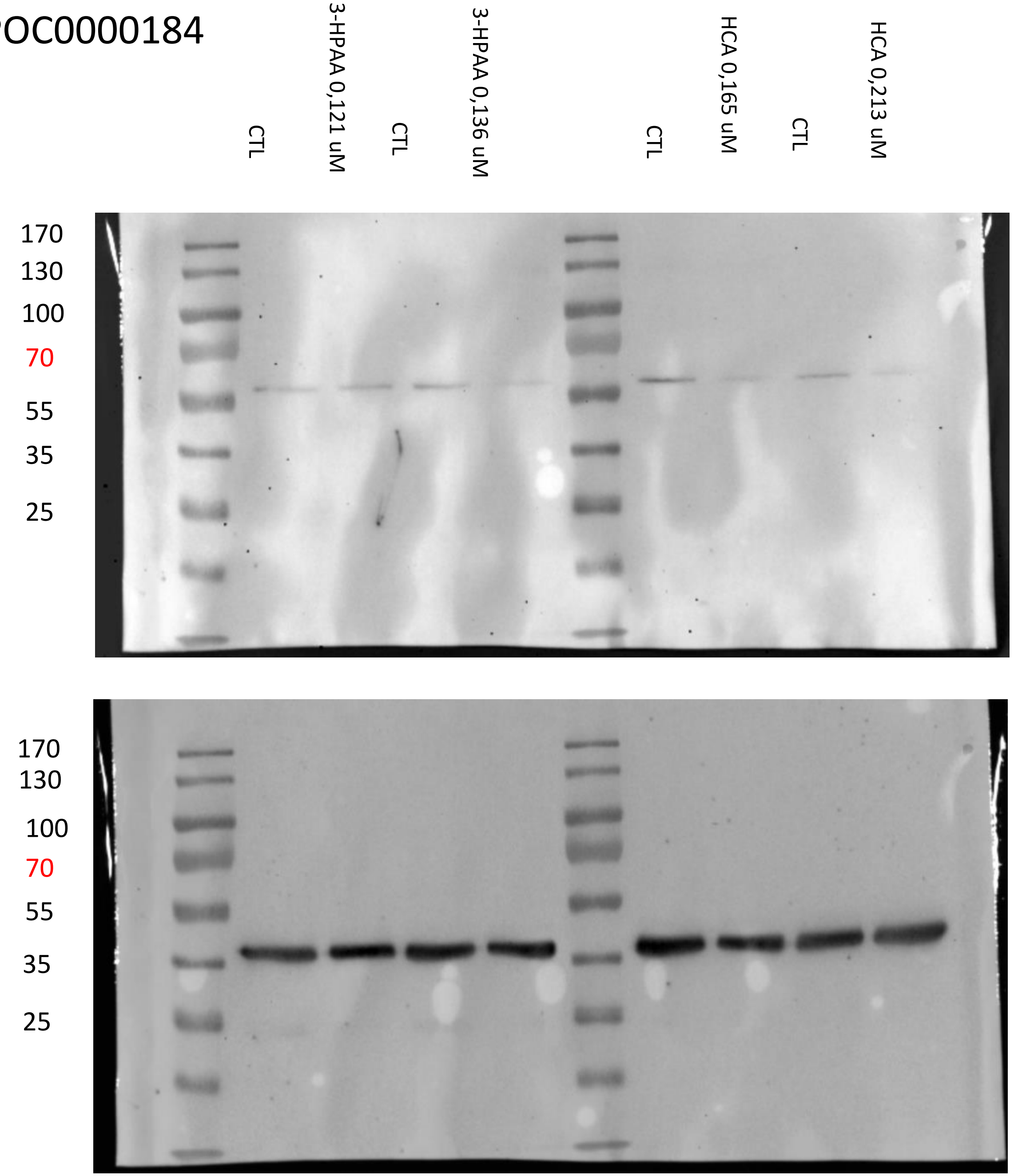

POC0000185

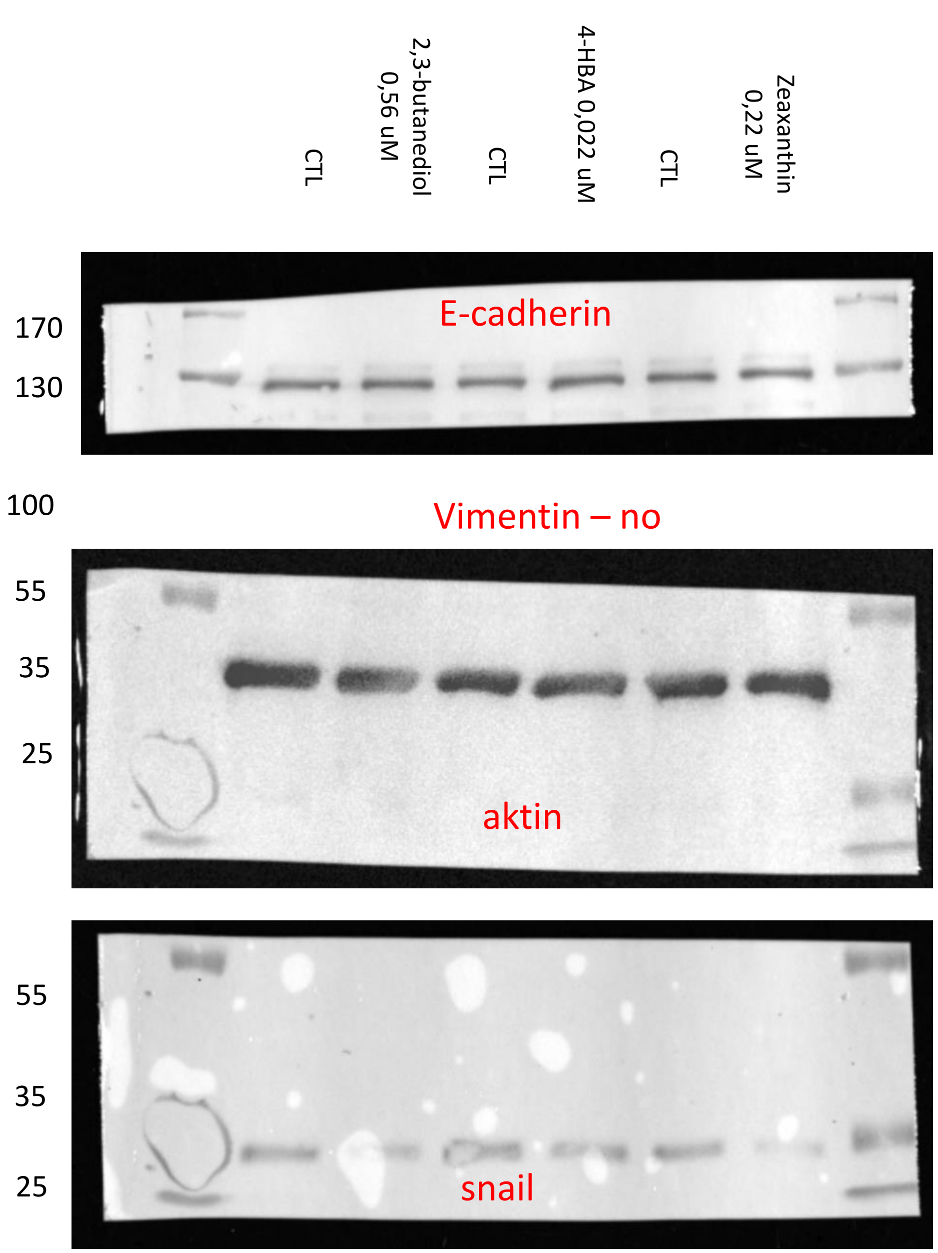

POC0000185

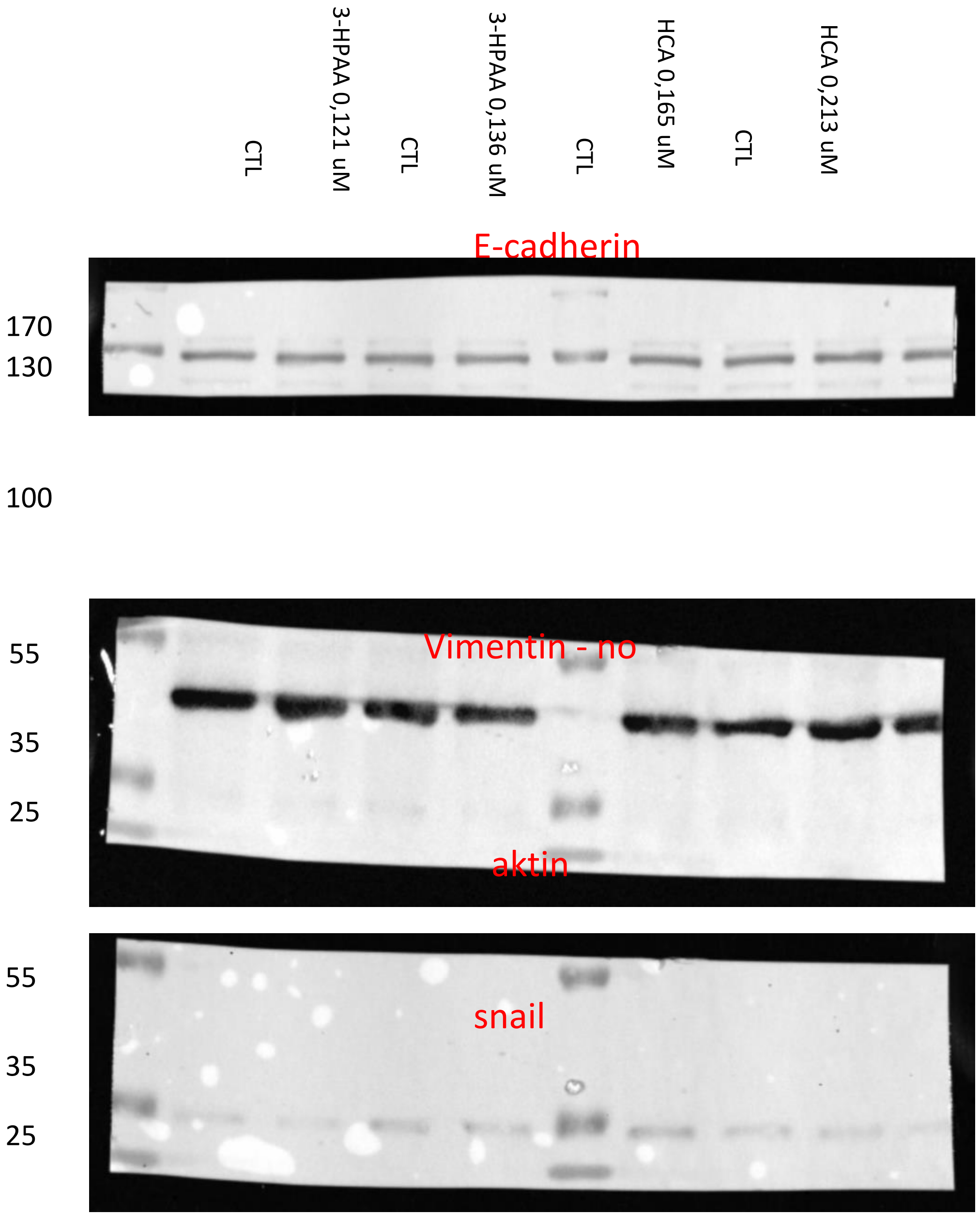

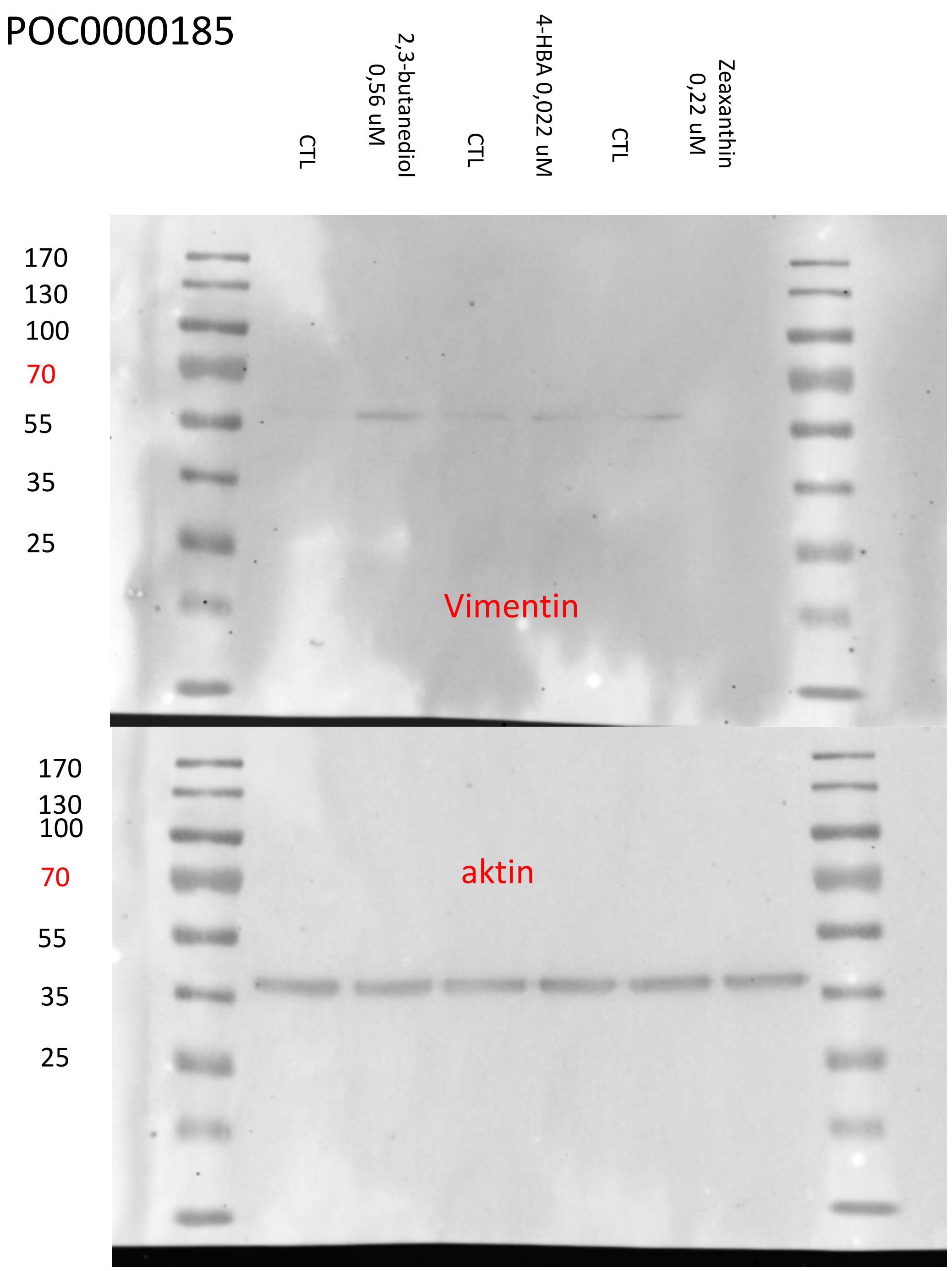
POC0000185
CTL
2,3-butanediol 0,56 uM
CTL
4-HBA 0,022 uM
CTL
Zeaxanthin 0,22 uM
170
130
100
70
55
35
25
Vimentin
170
130
100
70
55
35
25
aktin

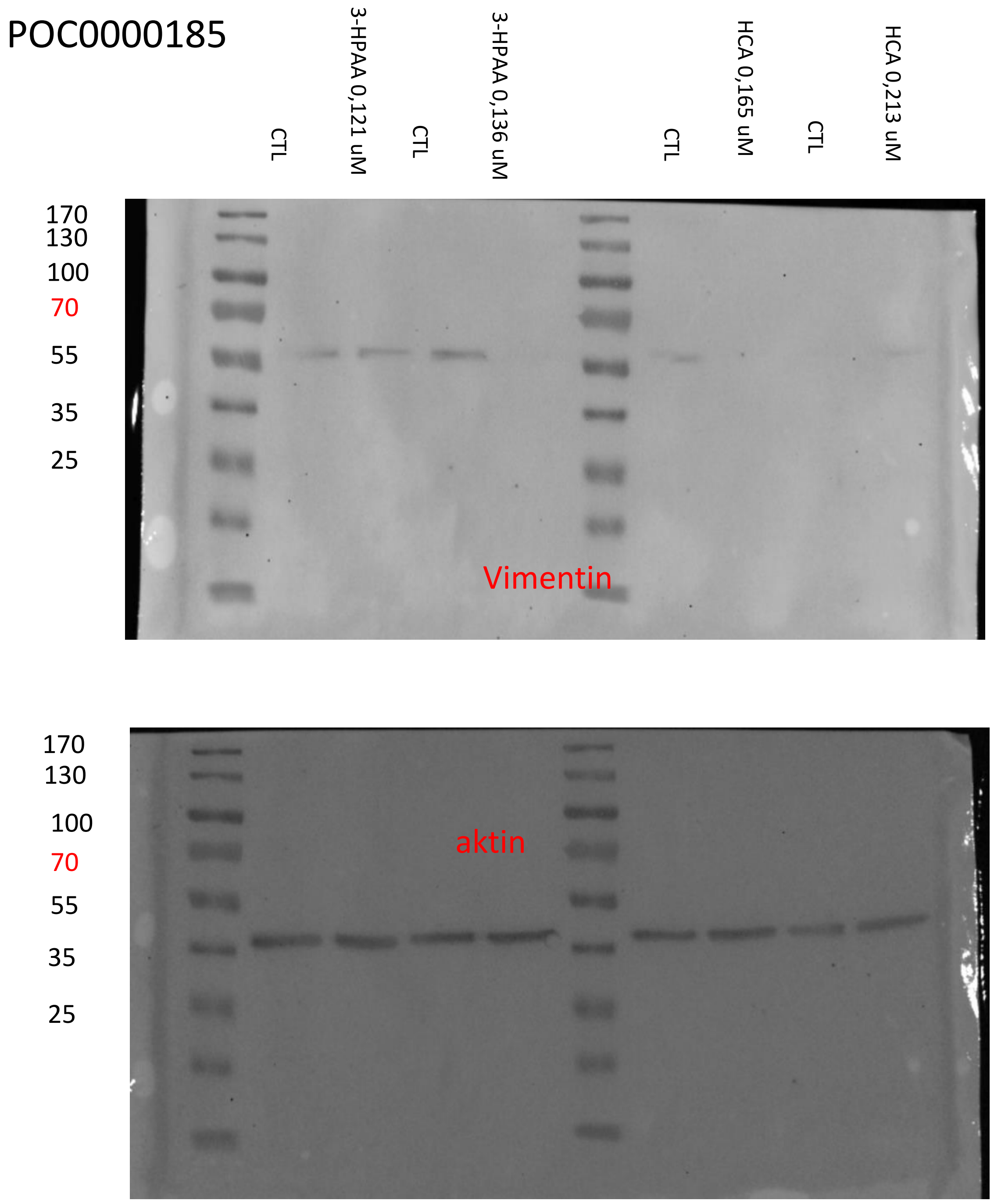
POC0000185
CTL
3-HPAA 0,121 uM
CTL
3-HPAA 0,136 uM
CTL
HCA 0,165 uM
CTL
HCA 0,213 uM
170
130
100
70
55
35
25
Vimentin
170
130
100
70
55
35
25
aktin
